# Neutrophils as critical orchestrators of chronic inflammation

**DOI:** 10.1038/s41423-025-01380-w

**Published:** 2026-01-13

**Authors:** Kaat Torfs, Gaël Vermeersch, Mieke Gouwy, Timothy Devos, Paul Proost, Sofie Struyf

**Affiliations:** 1 Laboratory of Molecular Immunology, Rega Institute, Department of Microbiology, Immunology and Transplantation, KU Leuven, Leuven, Belgium; 2 Department of Hematology, University Hospitals Leuven, Leuven, Belgium

**Keywords:** Neutrophil, Chronic inflammation, Innate immune response, Innate immunity, Neutrophils, Chronic inflammation

## Abstract

Neutrophils are the first key effector innate immune cells recruited toward inflammatory sites. Through the release of neutrophilic extracellular traps (NETs), the production of reactive oxygen species (ROS), degranulation and phagocytosis, neutrophils play a central role in the rapid elimination of invading pathogens. Recently, increasing attention has been given to the role of neutrophils in chronic inflammation, challenging the dichotomy between innate and adaptive immune responses. In chronic inflammatory conditions, neutrophils generally display a hyperinflammatory phenotype via dysregulated pathogen defense mechanisms. Excessive neutrophil activation may result in aberrant cell death, uncontrolled oxidative burst or NET formation and sustained release of inflammatory mediators such as proteases and inflammatory cytokines. Therefore, neutrophils contribute to the development of a sustained inflammatory environment and cause collateral tissue damage. In addition to their direct inflammatory effects, neutrophils further orchestrate inflammation and tissue remodeling by actively engaging in crosstalk with other cells within the immune microenvironment, such as endothelial cells, monocytes, platelets, and T and B cells. This review summarizes the current knowledge of the emerging role of neutrophils in the context of chronic inflammation. The key characteristics of neutrophils and their interactions with distinct cell types are discussed within the initial part of the review, whereas the second part focuses on their contributions to the pathophysiology of immune-driven diseases, including rheumatoid arthritis, atherosclerosis, inflammatory bowel disease, systemic lupus erythematosus, chronic obstructive pulmonary disease, and fibrotic disorders. Increasing knowledge on neutrophil behavior in the context of chronic inflammation may offer novel insights into disease pathology and, potentially, the identification of novel therapeutic targets.

## Introduction

Invading pathogens or endogenous signals may induce a self-limiting immunological response called “acute inflammation”. Acute inflammation is crucial for protecting against invading pathogens and initiating tissue repair. The Roman author Aulus Cornelius Celsus (30BC-38AD) first described the characteristics of acute inflammation and introduced its cardinal signs: *rubor* (redness)*, dolor* (pain)*, tumor* (swelling) and *calor* (warmth). Afterwards, Galen (129-c.216AD) added the fifth sign: “*functio laesa”* or “loss of function” [1].

When the resolution of inflammatory processes is disrupted, acute inflammation develops into a chronic state called “chronic inflammation”. The underlying cause may be the persistence of the initiating stimulus, impaired resolution of inflammation, or both. Many of the current leading causes of death are diseases characterized by an underlying state of chronic inflammation (e.g., atherosclerosis, autoimmune diseases or even cancer). Moreover, owing to population aging, many of these chronic inflammation-associated diseases are expected to become even more prevalent. As such, the role of innate immunity in age-related inflammation, called “inflammaging,” is a growing field of research [2].

Neutrophils represent the most abundant type of circulating leukocyte in the human body. As part of the innate immune system, neutrophils are among the first immune cells recruited to sites of inflammation and are therefore traditionally considered to be predominantly involved in acute inflammation. Nonetheless, especially during the last decade, the role of neutrophils in chronic inflammation and wound healing has increasingly been investigated and appreciated [3–5]. This review provides an in-depth discussion of the available knowledge on neutrophils in the context of chronic inflammation. For an in-depth review on the role of neutrophils in cancer and carcinogenesis, we refer to several excellent reviews that were published recently [6–8].

### Neutropoiesis and granule maturation

Underneath, we summarize current knowledge about neutrophil maturation and granule development. As previously described by Overbeeke et al. and Calzetti et al., we use the term “neutropoiesis” to describe the process of neutrophil maturation. Within the subtopic “granule biogenesis”, we focus on the development of different granules during neutropoiesis [9, 10].

### Neutropoiesis

In healthy individuals, an approximate number of 100 billion neutrophils are produced daily within the bone marrow. However, circumstances associated with “stress” or “emergency” neutropoiesis, such as sepsis, may increase neutropoiesis up to 10-fold. “Emergency neutropoiesis” refers to the situation in which neutrophil proliferation and differentiation are increased above basal levels, provoked by an acute event such as infection. During neutropoiesis, hematopoietic stem cells (HSCs) develop into neutrophil committed progenitors (NCPs), which are phenotypically characterized by the expression of CD64 and CD45RA. These NCPs evolve into promyelocytes through two distinct trajectories, among which CD34^+^CD45RA^−^ NCP1s and CD34^+^CD45RA^+^ NCP2s represent the earliest progenitors downstream of HSCs [10]. A recent report identified two previously undescribed NCP subtypes, NCP5 and 6, which immediately precede CD66b^+^CD11b^−^CD16^−^ promyelocytes. These populations can be distinguished on the basis of different mRNA and protein expression patterns. For example, NCP5s, and especially NCP6s, are characterized by relatively high expression patterns of α-defensins, which are only weakly expressed in earlier differentiation stages [11].

Although the process of neutropoiesis is far from completely understood, it is largely dependent on the cytokine granulocyte colony-stimulating factor (G-CSF). G-CSF is a hematopoietic cytokine that stimulates neutrophil production and hematopoietic stem cell mobilization. The G-CSF receptor (G-CSFR) is encoded by *GSF3R* and is a member of the type 1 cytokine receptor family, which also contains the receptor for interleukin-6 (IL-6) [12]. The central role of G-CSF in neutropoiesis is supported by the fact that G-CSF^−/−^ mice exhibit up to a 50% reduction in neutrophil precursors in the bone marrow and 60–70% in the peripheral blood [13]. In line with this, mice lacking G-CSFR are neutropenic, without affecting the counts of other white blood cells, erythrocytes or platelets. However, the presence of phenotypically normal neutrophils in G-CSF^−/−^ mice suggests the existence of other mechanisms of neutropoiesis independent of G-CSF signaling. Similarly, Liu et al. observed granulocyte production by plating bone marrow and splenic mononuclear progenitor cells in the presence of different cytokines, including IL-3, IL-6 and granulocyte/macrophage colony-stimulating factor (GM-CSF) [14]. Nonetheless, underlying neutrophil dysfunction induced by G-CSFR deficiency is conceivable, as G-CSF promotes neutrophil adhesion, CD11b upregulation and CD62L shedding in vitro [15, 16].

Evrard et al. proposed a classification of bone marrow-derived neutrophils into committed proliferative neutrophil precursors (preNeu) and nonproliferating neutrophils (i.e., an immature and mature subset) [17]. Transcriptomic and functional analyses have revealed a central role for the transcription factor C/EBPε, as genetic deletion (*Cebpe*^*−/−*^ mice) results in an almost complete block of neutrophil proliferation and differentiation beyond the stage of granulocyte‒macrophage progenitors (GMPs) [17]. CCAAT/enhancer binding protein-ε (C/EBPε) is a member of the C/EBP family of transcription factors that contains multiple proteins, including C/EBPε and C/EBPβ. While C/EBPε is required for terminal differentiation and maturation of neutrophilic granulocytes, C/EBPβ appears to be crucial for emergency neutropoiesis in response to cytokines or infections [18–20].

### Granule biogenesis

At least 3 subtypes of granules develop during different phases of neutropoiesis. Although these granules are described as different subtypes, they should be viewed as a continuum. Granule heterogeneity is the result of changes in protein expression during neutrophil maturation. Azurophilic (primary) granules are peroxidase-positive granules, indicating the presence of myeloperoxidase (MPO), and appear within the promyelocytic stage of development. Specific granules predominantly contain neutrophil gelatinase-associated lipocalin-2 (NGAL/LCN2) and lactoferrin. Tertiary granules contain high contents of gelatinases, including matrix-metalloproteinase 9 (MMP-9), and develop during both the metamyelocytic and banded stages of neutrophil maturation. Secretory vesicles, which are endocytic in origin, contain plasma proteins and develop during the final stages [21–23]. Compared with other granules, the secretory vesicles and granules formed during the last stages of neutrophil maturation are exocytosed more readily due to markedly different Ca^2+^-dependencies [24]. This increased sensitivity to a transient increase in Ca^2+^ is reflected in the increased density of vesicle-associated membrane proteins (VAMPs). VAMPs regulate the transport, docking, and fusion of vesicles and granules. These proteins are also referred to as “SNAREs”, which constitute distinct families of conserved membrane-associated proteins that facilitate membrane fusion [22, 25, 26]. Although neutrophils are widely accepted as important sources of immunomodulatory mediators, many questions remain concerning the exact production process of these components.

In addition to the release of prestored proteins, neutrophils perform de novo synthesis of multiple cytokines, including B-cell-activating factor (BAFF; discussed further in the “neutrophils and adaptive immunity” section), tumor necrosis factor (TNF)-related apoptosis-inducing ligand (TRAIL), the chemokines CXCL8 and CCL2 and the IL-1 receptor antagonist (IL-1Ra) [27, 28]. Even though the amount of a particular cytokine produced by one neutrophil may be lower than that released by other cells, neutrophil-derived cytokines may play an important role in patients because of the high abundance of neutrophils. By the release of either prestored or de novo synthetized immunomodulatory proteins at the site of inflammation, neutrophils are presumed (co-)orchestrators of local inflammatory reactions. However, as neutrophils are generally purified through density gradient or magnetic bead isolation techniques, there is an inherent risk that our current knowledge is biased by stimulation and/or degranulation of neutrophils during the isolation procedure and the presence of contaminating immune cells within purified samples. As neutrophils are RNA-poor cells, even small numbers of contaminating cells, particularly monocytes or macrophages, can result in significant skewing of expression patterns and thereby ambiguous conclusions [29]. We refer to an excellent article by Tecchio et al. for a more in-depth review of current evidence on cytokine production/expression by neutrophils [30].

## Neutrophil signatures and heterogeneity in inflammation

Neutrophils show adaptive properties and are able to change their phenotype and function in response to changing circumstances. These changing circumstances may include different cytokine or chemokine signatures, local production of reactive oxygen and nitrogen species (ROS/RNS), or local metabolites. Many researchers have tried to further unravel different neutrophil populations in health and disease. However, to date, no uniform classification of neutrophil heterogeneity exists.

Historically, neutrophils were purified from peripheral blood via density gradient centrifugation. Under inflammatory circumstances, at least two different populations can be distinguished after density gradient centrifugation of peripheral blood: normal-density neutrophils (NDNs) and low-density neutrophils (LDNs). These populations can be found on top of erythrocytes and within the mononuclear fraction, respectively. Unlike mature NDNs, the LDN fraction represents a heterogeneous population containing both mature and immature cell types. Among these are polymorphonuclear myeloid-derived suppressor cells (PMN-MDSCs) and low-density granulocytes, which have immunosuppressive and proinflammatory characteristics, respectively [31]. In addition, neutrophils are referred to as part of the “N1” or “N2-subset” on the basis of their effector functions and/or phenotypic characteristics. While “N1” corresponds with a proinflammatory phenotype, “N2” neutrophils are considered “anti-inflammatory” or often “protumoral” and are characterized by increased expression of MMP-9, vascular endothelial growth factor (VEGF), and upregulation of chemokine receptor CXCR4. Increasing evidence suggests that transforming growth factor-β (TGF-β) induces a protumoral phenotype [32–34]. However, there is often a significant translational barrier between preclinical murine models and clinical findings describing neutrophil populations in patients [33, 34]. Compared with humans, mice not only differ significantly in the percentage of circulating neutrophils but also in the molecular aspects related to neutrophil activation and signaling in mice. For example, one of the main neutrophil attractants and activators in humans, i.e., CXCL8 or IL-8, has no murine equivalent. Moreover, owing to the absence of a clear consensus on how to define different neutrophil subsets, researchers have to avoid a plethora of definitions. In addition, one can imagine that distinguishing different cell populations on the basis of density may result in aspecific and inaccurate conclusions and that NDNs may end within the LDN fraction depending on the exact blood handling procedures used [5]. Therefore, other groups aimed to provide more in-depth classifications of different neutrophil subtypes at either the transcriptomic or proteomic level. By using mass cytometry (CyTOF) and cell cycle-based analysis, Evrard et al. described three different neutrophil subsets within the bone marrow: a committed proliferative neutrophil precursor (preNeu), which consecutively differentiates into nonproliferating immature (CD101^-^) and mature (CD101^+^) neutrophils. Interestingly, while they are nearly absent from the circulation at baseline, immature neutrophils appear to migrate as efficiently as mature neutrophils toward sites of injury [17]. Although the exact role and consequences of this mobilization remain unclear, these immature cells might serve as a neutrophil reservoir ready to mobilize in times of inflammation [17]. In addition, Wigerblad et al. demonstrated via single-cell RNA sequencing (scRNA-seq) that, within peripheral blood, relatively immature (Nh0) cells evolve through a transitional Nh1 phenotype into one of two end points defined as Nh2 or Nh3 [35]. While the Nh2 phenotype is characterized by relative transcriptional inactivity, the Nh3 phenotype is characterized by high expression of type I interferon-inducible genes. By using Cellular Indexing of Transcriptomes and Epitopes by Sequencing (CITE-seq), they reported similar expression patterns of multiple surface proteins commonly used to characterize neutrophil subsets among Nh0-3 neutrophils (e.g., CD16, CD66b, CD10, and CD11b) [35]. These findings suggest that even OMICS methodology does not provide an unambiguous answer [17].

## Neutrophil effector functions in inflammation

### Reactive oxygen species (ROS)

**Fig. 1:**
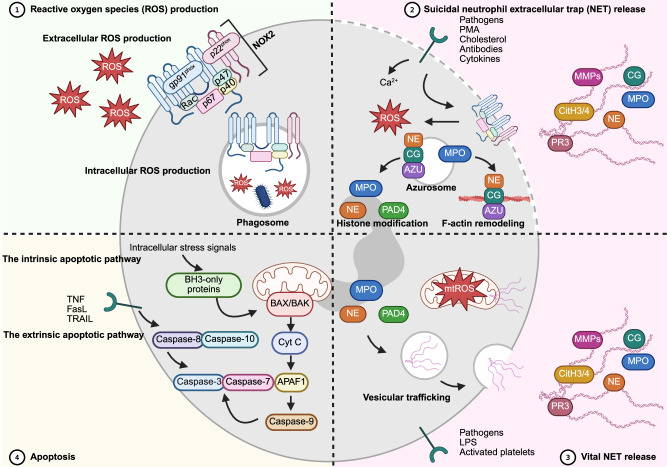
**Neutrophil effector functions in inflammation**. Panel 1 Neutrophils produce reactive oxygen species intracellularly, aiding in pathogen killing, and extracellularly, causing damage to the surrounding tissue. Crucial for ROS production is nicotinamide adenine dinucleotide phosphate (NADPH) oxidase 2 (NOX 2), which comprises 6 distinct subunits located at the cytosolic side of the plasma membrane. The membrane subunit flavocytochrome b558 contains p22^phox^ and the catalytic core gp91^phox^. Upon stimulation of neutrophils, the cytosolic subunits p47^phox^ (NCF1), p67^phox^ (NCF2), and p40^phox^ (NCF4) and the small GTPases Rac1 and Rac2 are recruited toward flavocytochrome b558 to form a functional electron transfer system. Panel 2 Neutrophils are able to expel web-like chromatin structures called neutrophil extracellular traps (NETs). The release of NETs (or NETosis) can occur in a NOX2-dependent or NOX2-independent manner, referred to as suicidal or vital NETosis, respectively. Suicidal, lytic or NOX2-dependent NET release can be initiated by various stimuli, such as pathogens, phorbol 12-myristate 13-acetate (PMA), cholesterol, antibodies and cytokines. These stimuli result in remodeling of chromatin and histone modification mediated by enzymes, including myeloperoxidase (MPO), neutrophil elastase (NE), and peptidylarginine deiminase (PAD4). In addition, among cytoplasmic enzymes, NE, cathepsin G (CG), and azurocidin (AZU) also cause remodeling of F-actin, which contributes to NET release. As a consequence, NETosis coincides with the expulsion of many proteins, including MPO, CG, NE, matrix metalloproteinases (MMPs), proteinase 3 (PR3) and citrullinated histones. Panel 3 Vital NETosis occurs independently of NOX2 through vesicular trafficking and is traditionally triggered by pathogens, lipopolysaccharide (LPS), or activated platelets. NETs containing mitochondrial DNA can be expelled in a nonlytic manner; however, contrary to the previous mechanisms, this process is dependent on NOX activity. Panel 4 Apoptosis is a tightly regulated form of programmed cell death and is dysregulated in many chronic inflammatory diseases. The intrinsic apoptotic pathway is induced by intracellular stress signals that activate B-cell lymphoma 2 (BCL-2) homology domain 3 (BH3)-only proteins. These proteins subsequently activate the BCL-2-associated X protein (BAX)/BCL-2 antagonist/killer (BAK) complex, causing mitochondrial membrane permeabilization and the release of cytochrome C within the cytoplasm. Cytochrome C, together with apoptotic protease-activating factor 1 (APAF1), cleaves procaspase-9, which subsequently results in the activation of procaspase-3 and procaspase-7 (i.e., effector caspases) and associated apoptotic cell death. The extrinsic apoptotic pathway is initiated by the binding of external factors, such as tumor necrosis factor (TNF), Fas ligand (FasL) and TNF-related apoptosis-inducing ligand (TRAIL). This pathway is mediated by the activation of caspases 8 and 10, which are in turn able to convert procaspase-3 and procaspase-7

Phagolysosomal production of ROS by neutrophils serves as a critical host defense mechanism against invading pathogens. However, excessive production of extracellular ROS can activate inflammatory signaling pathways and subsequently contribute to severe collateral tissue damage. A crucial protein for the production of extracellular ROS is the enzyme phagocyte oxidase or nicotinamide adenine dinucleotide phosphate (NADPH) oxidase 2 (NOX2) (Fig. 1). NOX2 consists of six distinct subunits, among which the flavocytochrome b558 domain is constitutively located in both the plasma and phagolysosomal membrane. Flavocytochrome b558 consists of two proteins: the catalytic core, gp91^phox^ (CYBB), and p22^phox^ (CYBA), which are required for structural integrity and function. The activation of neutrophils results in the assembly of an electron transfer system that is crucial for ROS production and is mediated by the recruitment of proteins called p47^phox^ [neutrophil cytosolic factor 1 (NCF1)], p67^phox^ (NCF2), p40^phox^ (NCF4) and the small GTPases Rac1 or Rac2. Electrons are first passed from NADPH to a flavin adenine dinucleotide (FAD) moiety and subsequently to Fe^3+^ within heme groups. These electrons are subsequently transferred to O_2_ molecules, which results in the development of superoxide radical anions [36, 37]. Finally, these highly reactive free radicals may be converted spontaneously, or mediated by the enzyme superoxide dismutase (SOD), into hydrogen peroxide (H₂O₂), which functions as a precursor for the formation of the highly microbicidal hypochlorous acid (HOCl) and hypothiocyanous acid (HOSCN) by MPO. In contrast, H₂O₂ may also be neutralized by enzymes, including catalase and glutathione peroxidase [38–40].

### Neutrophil extracellular traps (NETs)

NET formation, commonly referred to as NETosis, is a regulated form of cell death involved in the antimicrobial immune response. Upon stimulation, neutrophils may expel double-stranded DNA coated with modified histones and granule enzymes such as neutrophil elastase (NE), MPO, proteinase 3, cathepsin G, and MMPs into the extracellular environment (Fig. 1). In general, suicidal, lytic or NOX-dependent NET release is distinguished from vital, nonlytic or NOX-independent NET release. Suicidal NETosis, characterized by disruption of the plasma membrane, can be induced upon stimulation with molecules such as phorbol 12-myristate 13-acetate (PMA), cholesterol, antibodies, cytokines, or pathogens. Receptor activation causes a rise in intracellular calcium levels and triggers NOX activity. The production of ROS, particularly H_2_O_2_, drives the MPO-regulated translocation of NE, azurocidin and cathepsin G from the azurosome to the cytoplasm. These enzymes cause the remodeling of actin filaments. Furthermore, NE enters the nucleus, where it induces histone degradation and chromatin disassembly. In addition to NE, the enzyme peptidyl arginine deiminase type 4 (PAD4), which is also highly prevalent in neutrophils, facilitates chromatin decondensation by citrullinating histones H2A, H3 and H4. PAD4 is typically activated by increased intercellular Ca^2+^ ions or the production of ROS. In addition to suicidal NETosis, neutrophils can undergo vital NET release, which is independent of ROS production and characterized by the discharge of NET components by the plasma membrane through vesicular trafficking. Notably, NETs composed solely of mitochondrial DNA (mtDNA) can be expelled in a nonlytic manner; however, this process does require NOX activity. Important stimuli that induce vital NET release include pathogens, lipopolysaccharide (LPS) and activated platelets (through P-selectin/PSGL-1 interactions or soluble mediators) [41–44].

### Neutrophil cell death

#### Apoptosis

Apoptosis is a tightly regulated form of programmed cell death that plays a vital role in maintaining tissue homeostasis. The intrinsic apoptotic pathway is initiated by a variety of intracellular stress signals, such as DNA damage, growth factor withdrawal, stress within the endoplasmic reticulum, hypoxia and oxidative stress. These stressors activate proapoptotic B-cell lymphoma 2 (BCL-2) homology domain 3 (BH3)-only proteins, a subgroup of the BCL-2 family, which promote the activation of the BCL-2-associated X protein (BAX)/BCL-2 antagonist/killer (BAK) complex (Fig. 1). The activation of this complex induces oligomerization and subsequent pore formation in the outer mitochondrial membrane. Permeabilization of the mitochondrial membrane results in the release of cytochrome C within the cytoplasm, which can then bind to apoptotic protease-activating factor 1 (APAF1), resulting in the formation of an apoptosome capable of activating caspase-9. Caspase-9 is a crucial enzyme within the intrinsic apoptotic pathway, as it cleaves and activates the central effectors pro-caspase-3 and pro-caspase-7. The extrinsic apoptotic pathway is typically initiated by extracellular ligands such as TNF, Fas ligand (FasL) and TRAIL. These ligands bind to their respective death receptors, resulting in the activation of caspase-8 and caspase-10. These proteins can directly activate caspase-3 and caspase-7 and cleave BH3-interacting domain death agonist (BID), a BH3-only protein, into its truncated form, t-BID. Whether a cell undergoes apoptosis is determined by the balance between proapoptotic and antiapoptotic factors. Pro-apoptotic proteins include BH3-only members such as BID, whereas anti-apoptotic proteins include BCL-2, B-cell lymphoma-extralarge (BCL-xL), and myeloid cell leukemia 1 (MCL1) [45–47]. In chronic inflammatory diseases, e.g., chronic obstructive pulmonary disease (COPD) and rheumatoid arthritis (RA), neutrophil apoptosis is delayed, sustaining neutrophil activity [48, 49].

Apoptotic cells are characterized by membrane blebbing, cell shrinkage, vacuole formation, chromatin condensation, DNA strand breaks and other morphological changes, ultimately leading to the formation of apoptotic bodies [50]. The apoptotic bodies are removed by phagocytes, a process referred to as efferocytosis. To ensure the recruitment of phagocytes, apoptotic cells release chemotactic ‘find-me’ signals such as lysophosphatidylcholine (LPC). Additionally, the surface expression of certain ‘eat-me’ signals, particularly phosphatidylserine and calreticulin, which are recognized by their respective receptors on macrophages and dendritic cells, increases. This binding, with or without the involvement of bridging molecules, causes Rac1-mediated engulfment of these apoptotic bodies. Eventually, the fusion of phagosomes with lysosomes causes the degradation of cellular components by various digestive enzymes [51]. In autoimmune pathologies such as systemic lupus erythematosus (SLE), impaired efferocytosis causes neutrophils to undergo secondary necrosis, contributing to the release of autoantigens into the extracellular environment [52]. Furthermore, defective neutrophil uptake by phagocytes such as macrophages is related to impaired resolution of inflammation in COPD [53].

#### Ferroptosis

Ferroptosis is a alternatively controlled mechanism resulting in cell death characterized by increased intracellular iron levels. Accumulated Fe^2+^ ions participate in the so-called Fenton reaction, which results in the production of hydroxyl radicals through interactions with H₂O₂. These hydroxyl radicals damage cell membranes through lipid peroxidation, as they interact with polyunsaturated fatty acids and thereby contribute to cell death [54]. Importantly, glutathione peroxidase 4 (GPX4) and ferroptosis suppressor protein 1 (FSP1) were identified as protective enzymes that neutralize lipid peroxides. On the other hand, lysophosphatidylcholine acyltransferase 3 (LPCAT3) and arachidonate lipoxygenases (ALOXs), which are involved in the incorporation and oxidation of polyunsaturated fatty acids, respectively, promote ferroptosis. Ferroptotic neutrophils are characterized by mitochondrial dysfunction, as observed by electron microscopy [46].

#### Necroptosis and necrosis

Necroptosis is characterized by the release of damage-associated molecular patterns (DAMPs) into the extracellular environment and thus features both apoptosis and necrosis. Morphologically, necroptotic cells present characteristic features of necrotic cells, such as vacuole formation, enlargement of cell organelles, chromatin condensation (less pronounced), and membrane permeabilization. Necroptosis is mediated by activation of the TNF receptor, resulting in the formation of the necrosome, which consists of receptor-interacting protein kinase 1 (RIPK1) and RIPK3, which phosphorylate mixed lineage kinase domain-like (MLKL). This effector protein damages the cell membrane and activates the p38 mitogen-activated protein kinase (MAPK) pathway. In addition to TNF, other factors that induce necroptosis include the activation of toll-like receptors (TLRs) and GM-CSF priming followed by the ligation of the adhesion receptors CD44, CD11b, CD18, or CD15. X-linked inhibitor of apoptosis protein (XIAP) regulates necroptosis by inhibiting RIPK1. Furthermore, RIPK1-mediated inhibition of caspase-8 redirected the cell death process toward necroptosis rather than toward apoptosis. Notably, RIPK1- and/or MLKL-independent necroptosis of neutrophils has also been reported [46, 54].

In contrast to necroptosis, necrosis involves the passive, uncontrolled release of cellular debris, triggering inflammation and causing tissue damage. Physical and chemical stressors cause mitochondrial dysfunction through increased ROS production and excessive Ca^2+^ uptake, which leads to an osmotic imbalance. This causes swelling of the cell and its organelles, followed by irreversible rupture of the plasma membrane [54, 55].

#### Pyroptosis

Pyroptosis occurs through the inflammasome-dependent activation of caspase enzymes. These caspases are responsible for the cleavage of gasdermin proteins (GSDMs), which disrupt membrane integrity via pore formation. This results in cell lysis and facilitates the release of proinflammatory cytokines, including IL-1β and IL-18, and alarmins, such as high mobility group box 1 (HMGB1). In human neutrophils, the pyroptotic pathway can vary depending on the stimulus. For example, pyroptosis can be mediated by NLRC4 inflammasome activation, which leads to the caspase-1-dependent cleavage of GSDMD. Caspase-4 has also been shown to activate GSDMD and induce pore formation. In addition to caspase-driven pathways, NE has been reported to cleave GSDMD directly, representing an alternative mechanism contributing to pyroptotic cell death [47]. In chronic inflammatory diseases, neutrophil pyroptosis is involved in the exacerbation of inflammation. For example, the skin lesions of patients with psoriasis presented significantly increased expression of GSDMD, especially in neutrophils. GSDMD-dependent neutrophil pyroptosis results in excessive IL-1β release and thereby contributes to inflammation [56].

#### Autophagy

Autophagy is, as the name describes, a process in which the cell ‘eats’ itself. This process can be triggered by phagocytosis; internal stimuli, such as damaged organelles; and external stimuli, such as through the activation of TLRs. The preautophagosome is formed after double-membrane encapsulation of a certain target substance, a process regulated by autophagy-related proteins (ATGs). After maturation, the autophagosome fuses with a lysosome, causing enzymatic degradation of substances that can be used for cell recycling [54]. For example, in chronic asthma, neutrophil autophagy facilitates NET formation and thus contributes to airway inflammation [57].

## Neutrophils at the intersection of immunity

As part of innate immunity, neutrophils are the first responders attracted to the site of inflammation. In addition to directly contributing to the elimination of pathogens, it has become clear that neutrophils are able to influence the overall immune response. This can be achieved through multiple mechanisms, including cell‒cell interactions, or indirectly through the production/release of soluble mediators. Underneath, we provide a general, but nonexhaustive, overview of (in)direct neutrophil interactions with multiple cell types, including endothelial cells, thrombocytes, monocytes and adaptive immune cells.

### Neutrophil diapedesis

Acute and chronic inflammatory diseases, such as inflammatory bowel disease (IBD), are associated with increased infiltration of neutrophils. To infiltrate the tissue, neutrophils must extravasate from blood vessels through interaction with endothelial cells, a process called “diapedesis”. Traditionally, this process is divided into different phases, including selectin-mediated neutrophil rolling, activation of integrins, and arrest of cells. Neutrophil diapedesis is the result of a complex interplay between chemokines, glycosaminoglycans, and chemokine receptors; rearrangement of the cytoskeleton; interactions between adhesion molecules on neutrophils and endothelial cells; and changes at the endothelial level (e.g., the glycocalyx). Here, we briefly summarize the current knowledge and refer to other articles discussing these topics in greater depth [58–60].

**Fig. 2:**
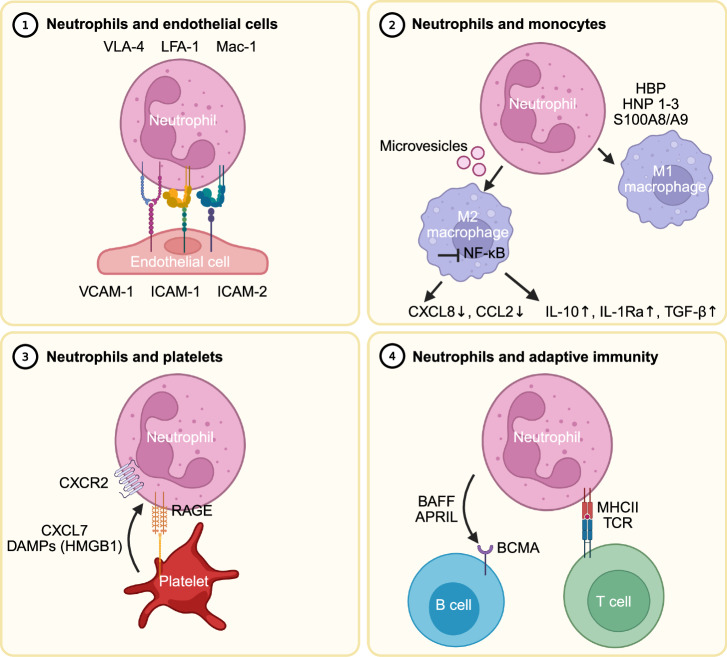
**Neutrophils at the intersection of immunity**. Neutrophils contribute to chronic inflammation through their ability to interact with various cell types. Panel 1 Neutrophil extravasation (called “diapedesis”) is mediated by multiple ligand‒receptor interactions with neutrophils and endothelial cells. Crucial mediators within this process include integrins on neutrophils [e.g., lymphocyte function associated antigen-1 (LFA-1 or CD11a/CD18 or αLβ2), macrophage-1 antigen (Mac-1 or CD11b/CD18 or αMβ2) and very late antigen-4 (VLA-4 or α4β1)] and adhesion molecules, containing immunoglobulin domains, on endothelial cells [e.g., intercellular adhesion molecule 1/2 (ICAM-1/-2) and vascular cell adhesion molecule 1 (VCAM-1)]. Panel 2 Neutrophils influence macrophage polarization toward either a pro- or anti-inflammatory phenotype (M1 or M2, respectively). Polarization toward the “M1 phenotype” is mediated by the release of neutrophil-derived peptides such as heparin-binding protein (HBP), human neutrophil peptides 1–3 (HNP1–3) and S100A8/A9. In contrast, anti-inflammatory reprogramming is mediated by the release of microvesicles by neutrophils and is characterized by the suppression of nuclear factor kappa light chain enhancer of activated B cells (NF-kB) signaling. As a consequence, the expression of proinflammatory cytokines is downregulated, while the expression of anti-inflammatory cytokines such as interleukin-10 (IL-10), IL-1Ra and transforming growth factor-β (TGF-β) is upregulated. Panel 3 Chronic inflammation results in a prothrombotic state called “immunothrombosis”, wherein mutual activation of thrombocytes, leukocytes and coagulation factors plays a central role. Crucial for this interaction is the binding of P-selectin glycoprotein ligand 1 (PSGL-1) on neutrophils with P-selectin on thrombocytes, which results in the release of chemokines [e.g., CXCL7] and danger-associated molecular patterns (DAMPs) [e.g., high mobility group box 1 (HMGB1)]. Panel 4 In addition to their role in initial immune responses, neutrophils influence the activity of adaptive immunity by interacting with B and T cells. B-cell-stimulating mediators produced by neutrophils include B lymphocyte stimulator (Blys), also known as BAFF, and “a proliferation-inducing ligand” (APRIL), which binds with B-cell maturation antigen (BCMA). In addition, neutrophils appear to possess antigen-presenting capacities [e.g., expression of major histocompatibility complex II (MHCII)] in specific circumstances and may thus contribute to the activation of the T-cell receptor (TCR) and steer adaptive immunity

Neutrophil adhesion to the endothelium in response to the activation of G protein-coupled receptors (GPCRs) for chemoattractants occurs through interactions between activated neutrophil integrins [e.g., lymphocyte function-associated antigen-1 (LFA-1 or CD11a/CD18 or αLβ2), macrophage-1 antigen (Mac-1 or CD11b/CD18 or αMβ2) and very late antigen-4 (VLA-4 or α4β1)] and their ligands on the surface of endothelial cells [e.g., intercellular adhesion molecule 1 (ICAM-1), ICAM-2 and vascular cell adhesion molecule 1 (VCAM-1)] (Fig. 2). Once attached, local polymerization of filamentous actine (F-actin), resulting in the development of lamellipodia, is initiated within neutrophils and allows directional migration toward a concentration gradient of chemokines. In contrast, it appears that the opposite side (i.e., “the uropod”) of the one that faces the chemotactic gradient is crucial within the initial phases of neutrophil crawling [59]. The uropod is characterized by dense clustering of P-selectin glycoprotein ligand 1 (PSGL-1), a glycoprotein ligand for P-selectin, and CD62L. Through PSGL-1, neutrophils scan for activated platelets within the circulation and allow their interaction with the “backside” of the neutrophil. Mice deficient in *Cdc42* (i.e., a Rho-GTPase required for neutrophil polarization and thus the development of the uropod) fail to form PSGL-1 clusters and display impaired crawling on endothelial cells [61]. This impaired crawling is potentially mediated by the altered distribution of Mac-1 or CXCR2, as mice deficient in PSGL-1 show mislocalization of both receptors. Once interaction with endothelial cells is established, diapedesis can occur paracellularly or transcellularly. Although poorly understood, neutrophil diapedesis is finalized by crossing pericytes (i.e., perivascular, multipotent cells enveloping the outer surface of the blood vessel wall and being crucial for vascular integrity) and the perivascular basement membrane. Within these final steps of diapedesis, mast cell-derived IL-17A appears to play a crucial role in promoting the expression of ICAM-1 and CXCL1 on pericytes in mice, thereby further mediating neutrophil crawling [59, 62, 63].

In addition to direct ligand‒receptor interactions between leukocytes and endothelial cells, the glycocalyx lining the surface of endothelial cells plays a central role in leukocyte diapedesis. The glycocalyx is an intravascular compartment that creates a dynamic barrier between the circulating blood and the vessel walls. It is composed of membrane-bound proteoglycans (i.e., long oligosaccharide chains covalently linked to core proteins) and glycoproteins (i.e., proteins with covalently attached shorter, branched oligosaccharide chains), creating a highly negatively charged endothelial surface and contributing to vascular integrity [64, 65]. Glycosaminoglycans may be maintained within the glycocalyx through both electrostatic interactions and oligomerization, thereby acting as a “chemokine sink”. On the other hand, according to this “cloud” model, the more hydrated parts of the glycocalyx could allow the development of a “chemokine cloud” providing soluble chemokines ready to interact with receptors on passing leukocytes [64]. Under inflammatory circumstances, neutrophils release a wide range of enzymes and reactive species that contribute to local or systemic glycocalyx damage. The release of neutrophil-derived components [e.g., cytokines, ROS/RNS, MMPs, MPO or NE] negatively affects glycocalyx integrity and thereby increases vascular permeability [66–70]. In addition, by degrading the outer parts of the glycocalyx, leukocytes gain access to increased amounts of chemokines and home within the “chemokine sink”, thereby promoting diapedesis [64, 71]. In addition to the effects of leukocytes and their derived products, the structure of the glycocalyx can be modified directly by chronic (inflammatory) diseases. For example, oxidized low-density lipoproteins (ox-LDL), present in atherosclerosis, as discussed further, contribute to the disruption of the endothelial glycocalyx [72]. Additionally, a small observational study of patients with diabetes mellitus indicated that inadequately controlled hyperglycemia (as defined by HbA1c ≥ 8%) is associated with the loss of the glycocalyx within the microcirculation. Although the underlying pathophysiological mechanisms have not been investigated, changes in shear stress or the development of advanced glycation end products are likely to contribute to disintegration of the glycocalyx [73, 74].

### Neutrophil‒monocyte interactions

Neutrophils are the predominant type of immune cell within the initial phase of inflammation and thereby contribute to the chemotaxis of other immune cells, among which monocytes are the most important. For example, in *Cebpε*^−/−^ mice, monocytes exhibit impaired migration. This *Cebpε* knockout animal model functions as a model for specific granule deficiency [75]. Indeed, lysates from patients with specific granule deficiency do not show monocyte chemotactic properties. Together, these data suggest that neutrophil granules may be important in modulating monocyte chemotaxis and accumulation in vivo [76].

In addition to monocyte attraction, neutrophils appear to influence the polarization of macrophages toward a pro- or anti-inflammatory phenotype (Fig. 2). While recent data indicate a continuum of active phenotypes, macrophages are classically considered to follow a dichotomous pattern of polarization toward the M1 or M2 phenotype [77]. The exact mechanisms by which neutrophils influence the macrophage phenotype are currently incompletely understood, but multiple mechanisms have been proposed whereby neutrophils further skew macrophages toward a pro- or anti-inflammatory phenotype, orchestrating the development or resolution of inflammation. For example, in a murine model (*Apoe*^−/−^) of atherosclerosis, neutrophils prime macrophages for cytokine production through the release of NETs (discussed further within the section “atherosclerosis”) [78]. Additionally, neutrophil-derived proteins such as heparin-binding protein (HBP), human neutrophil peptides 1–3 (HNP 1–3) and S100A8/A9 contribute to macrophage recruitment and facilitate activation toward a proinflammatory phenotype [79].

In contrast, through the release of microvesicles, neutrophils are able to induce anti-inflammatory reprogramming (i.e., polarization toward the “M2 phenotype”) within surrounding macrophages. This anti-inflammatory reprogramming is characterized by the suppression of nuclear factor kappa light chain enhancer of activated B cells (NF-κB) signaling within macrophages and consequently the suppressed release of inflammatory cytokines and chemokines, including TNF, CXCL8 and CCL2. In contrast, the levels of anti-inflammatory cytokines, such as IL-10, IL-1Ra and TGF-β, increase or remain unchanged [80–82]. Other authors have shown that ROS-producing neutrophils mediate the resolution of inflammation through the activation of AMP-activated protein kinase (AMPK). Similarly, within a study focused on liver repair, conditioned medium from neutrophils of mice with NOX2 deficiency (*Nox*2^−/−^) failed to upregulate hepatocyte growth factor (*Hgf)*, which is used as an anti-inflammatory marker, within Ly6C^hi^CX3CR1^lo^ monocytes/macrophages [83].

### Neutrophil‒platelet interactions

The presence of inflammation results in the development of a prothrombotic state (called “immunothrombosis”), wherein mutual activation of platelets, leukocytes (predominantly innate immune cells such as neutrophils) and coagulation factors plays a crucial role. Under physiological circumstances, the prothrombotic effects of inflammation find their origin in preventing the systemic spread of pathogens. However, during chronic inflammation, excessive activation of immunothrombosis, also called “thromboinflammation”, may occur and result in a systemic prothrombotic phenotype. Increased levels of circulating neutrophil‒platelet complexes have previously been described in patients with chronic inflammatory diseases such as RA, psoriasis, or SLE [84, 85]. The occurrence of leukocyte‒platelet complexes mediated by the expression of adhesion molecules on activated platelets (e.g., P-selectin interacting with PSGL1 on neutrophils) is crucial for this phenomenon [86]. Additionally, in murine models, activation of the P-selectin-PSGL1 axis contributes to NETosis and may thus further contribute to a prothrombotic state [87]. In addition to direct interactions between neutrophils and platelets, signaling molecules released by both cell types might further stimulate coagulation. For example, platelets release neutrophil-attracting chemokines, such as CXCL7, as well as DAMPs, which results in neutrophil activation (Fig. 2). For example, in systemic sclerosis (i.e., an autoimmune disease associated with the development of fibrosis throughout the skin or organs), IBD-activated platelets function as a source of HMGB1 [88, 89]. HMGB1 is a DNA chaperone that, under physiological circumstances, resides in the nucleus, where it plays a crucial role in maintaining the structure of chromosomes. Upon physiological stress, HMGB1 can be actively or passively released (e.g., by cell death) toward the extracellular niche, where it functions as a DAMP protein [90]. Interestingly, in murine models, the disulfide form of HMGB1 contributes to NETosis through interaction with receptor for advanced glycation end products (RAGE) on neutrophils, as mice deficient in RAGE (*Rage*^−/−^) presented significantly lower NETosis than did wild-type C57BL/6 mice [91]. Although not always reproducible, other groups have shown that the binding of HMGB1 to other receptors, including TLR4, also contributes to NETosis [91–93].

### Neutrophil interactions with adaptive immunity

As protagonists of innate immunity, neutrophils are first attracted to sites of inflammation. In addition to orchestrating initial immune responses, neutrophils also contribute to the activation and regulation of adaptive immune responses. The production and release of neutrophil-derived components play important roles in this process. Previous studies have shown that neutrophils function as a source of cytokines crucial for the survival, maturation, and differentiation of adaptive immune cells. As mentioned earlier, neutrophils can produce BAFF [also known as B lymphocyte stimulator (BLyS)] (Fig. 2). BAFF is a member of the TNF family of proteins and binds multiple receptors, such as B-cell maturation antigen (BCMA, expressed on B cells). Its expression is increased in multiple chronic inflammatory diseases, including SLE [94, 95]. BAFF appears to play a central role in B-cell maturation, as B-cell development in Blys-deficient (*Blys*^−/−^) mice tends to be halted in the T1 (CD21^−^, IgM^+^) stage of development [96]. Interestingly, within murine models, BAFF-producing neutrophils are present within the splenic plasma cell niche. BAFF is suggested to play a role in promoting the development of long-lived splenic plasma cells, which might contribute to resistance to B-cell-depleting therapies, as often observed within the context of autoimmune diseases [97]. In addition to BAFF, another neutrophil-derived cytokine called a proliferation-inducing ligand (APRIL; also known as TNF ligand superfamily member 13) influences B-cell function by promoting survival and proliferation. Like BAFF, APRIL binds B-cell receptors, including BCMA, and is secreted by neutrophils in chronic inflammatory diseases such as RA [98].

More extensively studied is the interaction between T cells and neutrophils, as neutrophils appear to acquire antigen-presenting cell (APC) properties in specific conditions. For example, while freshly isolated neutrophils express very little major histocompatibility complex II (MHCII, HLA-DR), their expression can be induced by in vitro exposure to multiple cytokines, including GM-CSF, interferon (IFN)-γ and TNF [99, 100]. In addition, immature and mature neutrophils from murine bone marrow are able to differentiate into hybrid cells with the properties of both neutrophils and dendritic cells when cultured with GM-CSF. These hybrid cells resemble traditional dendritic cells in morphology (e.g., oval-shaped nuclei and lamellar dendritic processes), surface phenotype [e.g., expression of major histocompatibility complex class II (MHCII), CD1d, CD80, and CD86…] and functionality (e.g., triggering T-cell proliferation, although with more limited capacity than traditional dendritic cells). In comparison, cocultures of human bone marrow cells (CD64^−^/CD14^−^ band cells) with GM-CSF, TNF-α and IL-4 resulted in the development of a hybrid population expressing dendritic cell markers (HLA-DR, CD1c and CD11c). However, their functional characteristics have not been investigated [101]. Similarly, increased expression of HLA-DR on neutrophils from the synovial fluid of patients with arthritis (i.e., rheumatoid arthritis, juvenile idiopathic arthritis or undifferentiated inflammatory arthritis) was demonstrated by RNA sequencing and mass cytometry [102]. The concept of autoantigen generation by local proteolysis, for example, through the release of MMPs by neutrophils and potential expression of MHCII, was initially called the “REGA model” (remnant epitopes generate autoimmunity) [103]. Nonetheless, the potential pathogenic role of hybrid cells currently remains largely unknown [104].

## The multifaceted role of neutrophils in chronic inflammatory diseases

### Rheumatoid arthritis (RA)

RA is a chronic autoimmune disease characterized by systemic and destructive synovial joint inflammation, with a global prevalence rate (age-adjusted) of ~224 per 100,000 individuals [105, 106]. The development of RA is multifactorial and involves both genetic and environmental predisposing factors. Although several disease susceptibility loci have been identified, monozygotic twin studies indicate a concordance of only 12–15%. Nonetheless, certain well-known genetic variants are associated with both the onset and severity of RA. These variants frequently occur within human HLA-DR alleles, such as HLA-DRB101 and HLA-DRB104, and are collectively referred to as ‘shared epitopes.’ This term encompasses specific amino acid sequences (e.g., QKRAA) located within the peptide-binding region of MHCII molecules, which are frequently observed in patients with RA [106].

**Fig. 3:**
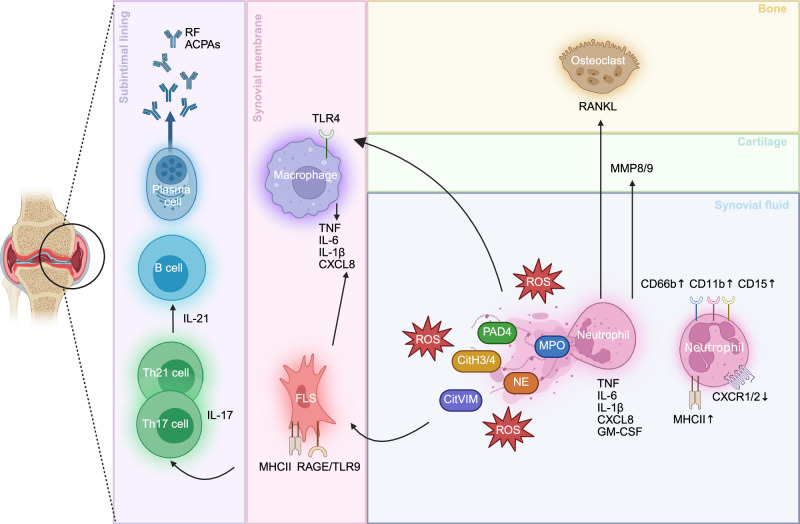
**Neutrophils in rheumatoid arthritis (RA)**. Neutrophils in the synovial fluid of patients with RA display a hyperinflammatory phenotype characterized by the upregulation of CD66b, CD11b, CD15 and major histocompatibility complex class II (MHCII) and the downregulation of CXC chemokine receptor 1/2 (CXCR1/2). They show increased protein expression of tumor necrosis factor (TNF), interleukin-6 (IL-6), IL-1β, CXC chemokine ligand 8 (CXCL8), and granulocyte macrophage-colony stimulating factor (GM-CSF). Neutrophils produce high levels of neutrophil extracellular traps (NETs), which are mediated by the activity of myeloperoxidase (MPO), neutrophil elastase (NE) and peptidylarginine deiminase 4 (PAD4), causing the release of citrullinated peptides, such as citrullinated vimentin (CitVIM) and citrullinated histones 3 and 4 (CitH3/4), into the extracellular environment. These citrullinated peptides are taken up by fibroblast-like synoviocytes (FLSs) through the receptor for advanced glycation end products (RAGE) and toll-like receptor 9 (TLR9) and are presented to T cells through MHCII. Subsequently, activated T helper 17 (Th17) and Th21 cells, which produce IL-17 and IL-21, respectively, stimulate the transformation of B cells into autoantibody-producing plasma cells. The key autoantibodies found in RA include rheumatoid factor (RF) and anti-citrullinated protein autoantibodies (ACPAs). ROS produced by neutrophils activate synovial macrophages (through TLR4) and FLSs, which in turn stimulate inflammation by producing high levels of TNF, IL-6, IL-1β and CXCL8. In addition to ROS and NETs, neutrophils within the synovial fluid of patients with RA release receptor activator of nuclear factor κB ligand (RANKL) and matrix metalloproteinases 8 and 9 (MMP8/9), resulting in osteoclast activation and cartilage degradation, respectively

In RA, multiple cell types and signaling molecules tend to play crucial roles. Memory T cells, located in the subintimal lining of the joint, activate B cells, causing them to differentiate into plasma cells (Fig. 3). These plasma cells secrete anti-citrullinated protein autoantibodies (ACPAs), rheumatoid factor (RF) or other autoantibodies commonly associated with RA. These autoantibodies can already be detected in the preclinical phase of RA and function as important diagnostic tools [106]. Synovial macrophages and fibroblasts produce several proinflammatory mediators (e.g., TNF, IL-6 and IL-1) and chemokines (e.g., CXCL8) [107, 108]. Local production of chemokines subsequently results in an abundant influx of other immune cells, including neutrophils, into the affected joint. Moreover, local processing of CXCL8 toward more potent proteoforms occurs within inflamed joints [109]. Upon activation, neutrophils release MMPs into the extracellular environment, resulting in extensive matrix degradation [110]. Neutrophils from the synovial fluid of patients with RA display an activated, hypersegmented phenotype with upregulation of adhesion molecules such as CD66b, CD11b and CD15 and downregulation of CXCR1/2. Within the synovial fluid of patients with juvenile idiopathic arthritis, neutrophils show increased HLA-DR, suggesting a potential role for these cells as antigen-presenting cells [111]. In addition, RA is often associated with extensive bone resorption driven by osteoclasts. Crucial within this process is the activation of osteoclasts by the receptor activator of nuclear factor kappa-B ligand (RANKL) expressed on neutrophils [106, 112, 113]. The R69-4 antibody, which binds type II collagen, diminished the presence of FcγRIII (CD16) on synovial fluid neutrophils in a collagen antibody-induced arthritis (CAIA) mouse model. The reduction in FcγRIII signaling impaired IL-1β self-amplifying neutrophil recruitment [114].

#### NET release in RA

Numerous studies have highlighted the critical role of NETs in the early pathogenesis of RA (Table 1). This involves an imbalance between increased NET release and impaired NET clearance (e.g., indicated by decreased serum DNase I activity) [115, 116]. Both plasma and serum levels of cell-free nucleosomes have emerged as highly sensitive and specific diagnostic biomarkers for RA [115, 117].

**Table 1:** Role of neutrophils and neutrophil-derived components in different chronic inflammatory diseases

Chronic inflammatory disease	In vitro*/m*urine (model) / human	Sample type/experimental set-up	Findings concerning neutrophils	References
RA	-In vitro	-Plasma and synovial fluid neutrophils from patients with RA	-Spontaneous NET formation	[115,116]
		-Healthy control human neutrophils	-Release of PAD2/4 upon NET release	[118**]**
		-Synovial fibroblasts from patients with RA	-NET uptake by synovial fibroblasts is mediated by TLR9	[121**]**
		-RA neutrophils	-IL-33 induces NET formation by RA neutrophils	[122**]**
		-RA synovial fibroblasts	-NETs are taken up by synovial fibroblast through TLR9 and induce IL-33 production	[122**]**
		-T cells harvested from the peripheral blood of patients with ACPA + RA	-Cryptic epitopes presented in the MHCII context activate ACPA + T cells	[125**]**
		-Incubation of osteoblasts with NETs	-NETs induce osteoclastogenesis through upregulation of the RANKL/OPG ratio (TLR4/9 mediated)	[129**]**
		-Co-culture of neutrophils with human gingival-derived stem cells (with or without COX2 gene silencing)	-The COX2-mediated inhibition of neutrophil NET release occurs through the activation of PKA which inhibits ERK	[130**]**
		-RA synovial fluid neutrophils	-Increased ROS production independent of priming	[49]
		-Healthy control human neutrophils	-Soluble immune complexes activate only primed human neutrophils	[49]
		-RA peripheral blood neutrophils	-Increased expression of anti-apoptotic proteins	[134**]**
		-RA synovial fluid neutrophils	-Neutrophils from the synovial fluid only undergo apoptosis when incubated with synovial fluid that has high levels of HIF-1α	[140**]**
		-RA synovial fluid neutrophils	-FAP-α activates PI3K, stimulating NOX2-dependent ROS production thereby inducing necroptosis	[151**]**
		-Peripheral blood RA neutrophils	-Decreased levels of NLRP3 with increased levels of caspase-1	[154**]**
		-Whole blood protein expression from patients with active RA	-Increased levels of NLRP3, ASC and caspase-1	[155**]**
		-Healthy control neutrophils	IL-18 primes neutrophils, upregulates expression of CD11b and FPR1, increases levels of intracellular calcium, activates the p38 MAPK signaling pathway and induces ROS production and NE release	[156**]**
		-Synovial fluid RA neutrophils	-Increased expression of LC3	[157**]**
		-Peripheral blood RA neutrophils	-Increased autophagosome formation	[157**]**
		-Peripheral blood RA neutrophils	-Increased autophagosome formation	[158**]**
	-Murine (CIA)	-X-ray and H&E staining of knees and ankle joint	-Treatment with a therapeutic ACPA inhibits NET-induced tissue damage	[120**]**
	-Murine (HLA-DRB1*04:01)	-Quantification of serum ACPA levels	-NETs are taken up and presented by synovial fibroblasts to T cells eventually resulting in the generation of ACPAs	[121**]**
	-Murine (CAIA)	-Modified ELISA to measure citH3-DNA in the serum of these mice	-IL-33 stimulates NET release	[122**]**
	-Murine (*Tlr2*^-/-^)	-qPCR and flow cytometry	-NET histones stimulate the release of IL-17 by Th17 cells through TLR2	[123**]**
	-Murine (HLA-DRB1*04:01)	- The tibiofemoral compartment (TRAP staining)	- Carbamylated NETs induced osteoclastogenesis	[127**]**
	-Murine (*Micl*^−/ −^)	-Immunofluorescence and sytox green staining	-Increased NET formation in these mice due to loss of negative feedback through MICL	[128**]**
	-Murine (AIA)	-Histology and CT (bone loss)	-NETs induce osteoclastogenesis	[129**]**
	-Murine (STIA)	-Immunofluorescence, clinical score, gene silencing and flow cytometry	-Human gingival-derived mesenchymal stem cells release prostaglandin E2 which decreases NET formation in neutrophils.	[130**]**
	-Mice (*Ncf1*-/-)	-Neutrophil recruitment, gene expression, flow cytometry	-Mice deficient in NOX2 showed higher levels of *Cxcl2, Cxcl3* and *Cxcl10* as well as *Mmp3* alongside decreased expression of PD-L1	[132**]**
	-Murine (CIA)	-Arthritis score, paw swelling, recruited neutrophils	-Inhibition of NOX2 resulted in the development of arthritis	[133**]**
	-Murine (CIA)	-Arthritis severity	-In early RA, inhibition of TIM-4 worsened arthritis	[144**]**
	-Murine (CAIA)	-Arthritis severity	-In established RA, inhibition of TIM-4 improved arthritis	[144**]**
	-Murine (*Tyro3*^-/-^, *Axl* ^-/-^, *Mertk* ^-/-^)	-Levels of inflammatory cytokines and arthritis assessment	-KO of Tyro3 improved inflammation and decreased the levels of inflammatory cytokines, opposite findings for Axl and Mertk KO mice	[143**]**
	-Murine (*Elmo1*^-/-^)	-Arthritis assessment, bone degradation and neutrophil recruitment	-ELMO1, involved in efferocytosis, promotes inflammatory arthritis	[145**]**
	-Murine (CIA)	-Loss of Rac1 function	-Rac1, which works downstream of ELMO1 also promotes arthritis	[144**]**
	-Murine (STIA)	-Flow cytometry and arthritis scores	-Less neutrophil infiltration and reduced inflammation upon treatment with anti-SIRPa agonistic antibody	[146**]**
	-Murine (*Ifng*^-/-^)	-Measurment of necroptosis markers and severity of inflammation	- Increased levels of MLKL, RIPK1, and RIPK3, along with more severe joint damage and hyperinflammation150	[152**]**
	-Human	-Synovial fluid	-Increased levels of IL-33	[122**]**
			-Frequency of albumin carbamylation correlates with synovial fluid MPO activity	[126**]**
			-carbamylated NET proteins are increased	[127**]**
		-Plasma	-Cell-free nucleosomes as a potential diagnostic biomarkers	[115**]**
			-Carbamylated NET proteins are increased	[127**]**
		-Serum		[117**]**
		-Synovial fluid	-Cell-free nucleosomes as a potential diagnostic biomarkers	[128**]**
		-Synovial fluid	-Presence of anti-MICL antibodies	[134**–**137]
		-Synovial fluid	-Increased levels of G-CSF, GM-CSF, IL-1β, TNF, IFN-α and IFN-γ	[138,139]
		-Peripheral blood	-Adenosin and lactoferrin are upregulated and regulate apoptosis	[147**]**
				[148**]**
		-Serum	-Ferrous ion levels correlating with DAS28 scores	[153**]**
		-Synovial fluid	-Decreased levels of glutathione and GPX4	[158**]**
			-Increased levels of IL-18 and caspase –1	
			-Increased levels of IL-6, IL-10, CCL2 and CXCL8, inducing autophagy	
Atherosclerosis	-Murine (*Apoe*^−/−^)	-Intravital imaging	-Neutrophil infiltration during early atherosclerosis	[163**]**
	-Murine (*Apoe*^−/−^)	-Intravital imaging	-High neutrophil counts in rupture prone lesions	[164**]**
	-Murine (*Lysm*^gfp/gfp^*Apoe*^-/-^)	-Intravital imaging	-Luminal adherence and NETs release at atherosclerotic prone regions	[168**]**
	-Murine (ApoE/PR3/NE^−/−^)	- Intravital imaging, ELISA,..	- NET-mediated priming of macrophages to produce inflammatory cytokines	[78]
	-Human	- Plasma	-Increased S100A12 correlating with increased risk of major cardiovascular events	[160**]**
		-Immunohistochemistry carotid plaques	-High neutrophil counts in rupture prone atherosclerotic lesions	[165**]**
		-Immunohistochemistry	- High NETs count in complicated coronary plaque segments	[172**]**
IBD	-In vitro	- Incubation of CCD-18Co cells (human fibroblast cell line derived from colon tissue) with NGAL	- NGAL mediated induction of profibrotic phenotype in CCD-18Co cells (data also validated in mice with DSS-induced colitis)	[177**]**
		- Incubation of butyrate with neutrophils from patients with IBD	-Reduced production of inflammatory mediators (e.g. IL-6, TNF, S100A8/A9, LCN2,…)	[199**]**
	-Murine (PAD4^-/-^)	- Western blot,…	-PAD4 mediated citrullination of CKMT1 exacerbating mucosal inflammation in IBD	[181**]**
	-Human	-Faecal samples	- S100A8/A9 used as biomarker for disease activity	[174**]**
		- Immunohistochemistry colon biopsies	-Increased levels of PAD4	[180**]**
		- Blood and biopsies	-Correlation between MPO concentrations and disease activity	[182**]**
			-Neutrophil-dominant inflammation within corticoid-resistance	[183**]**
			-Increased expression of OSM (mRNA) correlates with poor response to anti-TNF therapy.	[185**]**
			- Potential protective role of CD177+ neutrophils (e.g. rol in maintaining mucosal integrity)	[188**]**
SLE	In vitro	-HC neutrophils stimulated with SLE plasma samples	-TLR8 is important for the recognition of RNA-containing immune complexes by neutrophils in SLE	[206**]**
		-Neutrophils from patients with SLE	-Neutrophils and platelets form TLR-7-dependent complexes, inducing NETosis	[195**]**
		-Neutrophils from blood of patients with SLE	-Normal density neutrophils express more CXCL10 and MMP8 but lower CD66b and release less NETs	[208**]**
		-Healthy control neutrophils incubated with SLE serum	-Circulating immune complexes in the serum are responsible for ROS production	[211**]**
		-Neutrophils from the peripheral blood of patients with SLE	-Loss of the inhibitory Gal1- VSTM1 loop in patients with SLE	[216**]**
		-Incubation of HC neutrophils with SLE serum stimulated with GM-CSF	-Decreased apoptosis	[216**]**
		-Incubation of HC neutrophils with SLE serum containing caspase-8 and caspase-9 inhibitors	-Decreased apoptosis	[54,201,219]
		-Neutrophils harvested from patients with SLE incubated with SLE serum	-Increased expression of autoantigens on the cellular surface which bind TLR3,8 and 9 on PBMCs	[54]
		-Neutrophils incubated with microparticles from SLE patients	-NETosis was induced	[221,222]
		-Dendritic cells incubated with microparticles from SLE patients	-Release of IL-6, TNF and IFN-α	[224**]**
		- Incubation of neutrophils with SLE serum	-FcγR-mediated activation of caspases 1 and 11, activating gasdermin D	[225**]**
		-In vitro incubation of neutrophils with SLE serum	-Expression of GPX4 is regulated through CREM-α	[226**]**
				[227**]**
	-Murine (MRL/lpr)	-IV administration of CXCL5	-IV administration of CXCL5 reduced neutrophil proliferation, activation and recruitment	[210**]**
	-Murine (*Ncf1m1J*)	-Gene expression, ELISA, flow cytometry	-Impaired ROS production, increased expression of IFN type I-regulated genes and increased levels of autoantibodies	[217**]**
	- Murine (MRL/lpr)	-Neutrophils isolated from these mice	-SLE IgGs stimulate neutrophil apoptosis through FcγRIII and the upregulation of FasL	[220**]**
	-Human	-Whole blood and plasma of patients with SLE	-Improved neutropenia, decreased apoptosis and decreased NETosis	[228**]**
		-Blood transcriptomics	-Treatment with belimumab reduced neutrophil counts and activation, neutrophil degranulation was a predictor of response to belimumab treatment	[229**]**
		-Peripheral blood	-Lower levels of CXCL5 compared to HCs	[210**]**
		-Peripheral blood	-Increased levels of oxidized Gal1 and reduced glutathione	[216**]**
		-Peripheral blood	-NETs or NET-related genes HMGB1, ITGB2 and CREB5 as biomarkers	[202**–**205]
		-SLE patients carrying a mutation in the NCF1 gene	-Lower levels of CXCL5 compared to HCs	[210**]**
		-Serum	-Impaired ROS production related to SLE development	[218**]**
		-Macrophages from SLE patients	-Increased levels of FasL, TRAIL and decreased levels of GM-CSF	[54,201,219]
		-Serum	-Reduced CD44 expression and reduced phagocytotic capacity, correlating with anti-nuclear antibodies and disease activity	[54,223]
			-Increased levels of TAM receptors	[54,223]
COPD	-Murine (C57BL/6 J; FVB/N exposed to ciragette smoke)	-Microscopy	-Decreased efferocytosis	[233**]**
	-Murine (C57BL/6 J)	-ELISA	- Increased NE	[252**]**
	-Murine (cGAS^-/-^/TLR9^-/-^)	-Immunofluorescence microscopy	- Increased NETs / inducing NETs mediated inflammation	[254**]**
	-Murine (Balb/c mice exposed to ciragette smoke)	-BALF	- Increased MMPs (e.g. MMP9)	[248**]**
	-Murine (IKTA)		-NE mediated inhibition of elastic fiber assembly in fibroblasts	[255**]**
	-Human	-Exhaled breath condensate	-Increased NE	[234**–**237]
	-Human	-Serum	-Increase of MMPs (e.g. MMP-9)	[249**–**251]
		-Sputum	-Increased NETs	[238**]**
		-Sputum	-Increased neutrophil and CXCL8 counts	[234**–**237]
		-Sputum	-Increased amount of PGP matricryptin (i.e. suggested to interact with CXCR1/2).	[284**]**
Fibrotic diseases (IPF)	-In vitro	- Co-culture of MMP-9 with human lung embryonic fibroblasts (MRC-5)	- Activation of TGF-β and induces expression of αSMA in fibroblasts	[275**]**
	-Human	-BALF	-Increased NE & NETs	[265,266]
		-BALF	-Increased S100A9 counts	[256**]**
		-BALF & serum	-Increased CXCL8 counts	[258,259]
Fibrotic diseases (MF)	-Human	-Peripheral blood neutrophils	-Increased basal ROS produced by JAK2+ neutrophils	[297**]**
		-Plasma	-Increased NE	[299**]**
		-Serum	-Increased MMP9/TIMP1	[300**]**
		-Peripheral blood Neutrophils	-Increased CD24 expression on neutrophils in JAK2+ neutrophils (e.g. resulting in decreased efferocytosis)	[301**]**
		-Bone marrow biopsies	-Increased emperipolesis of neutrophils by megakaryocytes	[304**–**306]

*α- SMA* α-smooth muscle actin, *ACPA* anti-citrullinated protein antibodies, *AIA* antibody-induced arthritis, *ApoE* apolipoprotein E, *ASC* apoptosis-associated speck-like protein containing a CARD, *AXL* axl tyrosine-protein kinase receptor, *BALF* bal fluid, *CAIA* collagen antibody-induced arthritis, *CCL* CC chemokine ligand, *cGAS* cyclic GMP-AMP synthase, *CIA* collagen-induced arthritis, *CKMT1* mitochondrial creatine kinase 1, *COX* cyclooxygenase, *COPD* chronic obstructive pulmonary disease, *CREB5* cAMP responsive element binding protein 5, *CREM-α* cAMP-responsive element modulator-α, *CXCL* CXC chemokine ligand, *DAS* disease activity score, *DSS* dextran sodium sulfate, *ELISA* enzyme-linked immunosorbent assay, *ELMO1* engulfment and cell motility protein 1, *ERK* extracellular signal-regulated kinase, *FAP-α* fibroblast activation protein-α, *FasL* Fas ligand, *FcγR* Fc-γ receptor, *FPR1* formyl peptide receptor 1, *Gal-1* galectine-1, *G-CSF* granulocyte-colony stimulating factor, *GM-CSF* granulocyte macrophage-colony stimulating factor, *GPX4* glutathion peroxidase 4, *H3* histone 3, *HIF-1α* hypoxia-inducible factor-1α, *HLA* human leucocyte antigen, *HMGB1* high mobility group box 1, *IFN* interferon, *IgG* immunoglobulin G, *IL* interleukin, *IPF* idiopathic pulmonary fibrosis, *ITGB2* integrin beta 2, *JAK* janus-kinase, *LC3* microtubule-associated protein 1 light chain 3, *LysM* lysine motif, *MAPK* mitogen-activated protein kinase, *MERTK* mer tyrosine kinase, *MHC* major histocompatibility complex, *MICL* myeloid inhibitory C-type lectin-like receptor, *MLKL* mixed lineage kinase domain-like, *MMP* matrix metalloproteinase, *MPO* myeloperoxidase, *MF* myelofibrosis, *MPN* myeloproliferative neoplasm, *NCF* neutrophil cytosolic factor, *NE* neutrophil elastase, *NET* neutrophil extracellular trap, *NGAL* neutrophil gelatinase-associated lipocalin, *NLRP3* NLR family pyrin domain containing 3, *OPG* osteoprotegerin, *OSM* oncostatin M, *PAD* peptidylarginine deiminase, *PBMCs* peripheral blood mononuclear cells, *PD-L1* programmed death-ligand 1, *PI3K* phosphatidylinositol 3-kinase, *PKA* protein kinase A, *PR3* proteinase 3, *RAC* small GTPase of the rho family, *RA* rheumatoid arthritis, *RANKL* receptor activator of nuclear factor kappa-B ligand, *RIPK* receptor-interacting protein kinases, *ROS* reactive oxygen species, *S100A12* S100 calcium-binding protein A12, *SIRP-α* signal-regulatory protein-α, *SLE* systemic lupus erythematosus, *STIA* serum-transfer-induced arthritis, *TAM* tyro3 axl mertk, *TGF-β* tumor growth factor-β, *Th* T helper, *TIM-4* T-cell immunoglobulin and mucin domain-containing protein-4, *TIMP* tissue inhibitor of metalloproteinases, *TLR* toll- like receptor, *TNF* tumor necrosis factor, *TRAIL* TNF-related apoptosis-inducing ligand, *TRAP* tartrate resistant acid phosphatase, *TYRO3* tyro3 protein tyrosine kinase, *VSTM1* V-Set and transmembrane domain containing 1

It is generally accepted that the citrullination of proteins contributes to the development of autoantigens and thereby autoimmunity in rheumatoid arthritis. Human neutrophils undergoing NETosis in vitro release PAD2/4 enzymes into the extracellular environment. These proteins are believed to be responsible for the citrullination of extracellular proteins such as fibrinogen and collagen [118, 119]. The administration of therapeutic ACPAs, which specifically recognize citrullinated histones H2A and H4, has recently been shown to inhibit NET-mediated cartilage and bone damage in a collagen-induced arthritis (CIA) mouse model because of decreased NET extrusion and/or increased NET clearance by macrophages [120]. NETs are internalized in a RAGE/TLR9-dependent manner by synovial fibroblasts, which are stimulated by NET-derived IL-17B to express MHCII, as shown in vitro. In vivo experiments with HLADRB1*04:01 transgenic mice confirmed the generation of serum ACPAs upon stimulation of synovial fibroblasts with NETs [121]. IL-33, which is upregulated in the synovial fluid of patients with RA, maintains a positive feedback loop by enhancing extracellular trap release and ROS production by neutrophils, which in turn activates synovial fibroblasts through TLR9, resulting in the production of more IL-33 in vitro. The pathological role of IL-33 was confirmed in CAIA mice: treatment with IL-33 led to more severe inflammation and increased NET formation in the joints [122]. More recently, NET histones themselves were shown to activate Th17 cells through TLR2 in *Tlr*2^−/−^ mice [123]. The Th21 T-cell subset plays a key role in the activation of B cells, promoting their differentiation into plasma cells that produce ACPAs. These highly specific ACPAs are detectable in ~80–90% of patients with RA and, remarkably, in 20% of their immediate family members. A positive feedback loop is established wherein immune complexes containing ACPAs and RF stimulate further NET release by neutrophils [124]. However, recent in vitro and in silico findings have proposed the concept of cryptic epitopes, suggesting that citrullination modifies peptide presentation in the MHCII context rather than simply generating citrullinated epitopes. These cryptic epitopes activate T cells harvested from ACPA-positive RA patients [125].

Furthermore, proteomic analysis revealed that increased levels of carbamylated proteins in the synovial fluid of patients with RA and synovial fluid MPO activity were correlated with albumin carbamylation [126]. Similarly, high levels of carbamylated NET histones were detected in the plasma and synovial fluid of patients with RA. In line with patient data, carbamylated NET histones activate TLR4 and induce osteoclastogenesis in HLA-DRB1*04:01 mice [127].

Other components within NETs may also contribute to disease progression. NET-derived DNA binds the myeloid inhibitory C-type lectin-like (MICL) receptor, which further inhibits neutrophil activation. Owing to the presence of anti-MICL receptor antibodies in the sera of patients with RA, this process is likely dysregulated in RA, as confirmed in a *Micl*^−/−^ mouse model [128]. NETs drive in vitro osteoclast formation by binding TLR4 and TLR9 and subsequently upregulating RANKL and downregulating osteoprotegerin. This finding was confirmed in an antigen-induced arthritis (AIA) mouse model in which treatment of NETs with DNases decreased bone resorption [129]. Treatment of serum transfer-induced arthritic (STIA) mice with human gingival-derived mesenchymal stem cells alleviated arthritis and suppressed NET formation via cyclooxygenase 2 (COX2)-mediated signaling. In vitro studies further confirmed that the PGE2–PKA–ERK axis plays a pivotal role in mediating this reduction in NET formation [130].

#### ROS in RA

In RA, neutrophils are stimulated by immune complexes through FcγRIIa and FcγRIIIb, leading to elevated production of ROS. As a result, they contribute to the inflammatory environment by expelling proteases from their granules, which in turn causes further degradation of the extracellular matrix and the production of ROS, which activate other inflammatory cells, such as synovial fibroblasts and T cells [44].

Neutrophils harvested from RA synovial fluid are primed to produce ROS, whereas peripheral blood neutrophils from patients with RA require priming with TNF. The genes predicted to be involved in priming in vivo include IFN-γ, G-CSF, TNF, IL-1β and IL-6 (Table 1). In vitro incubation experiments revealed that soluble immune complexes present in the synovial fluid activate ROS production only in primed human neutrophils, whereas insoluble immune complexes, also present in the synovial fluid, stimulate both unprimed and primed human neutrophils [49, 131]. Interestingly, the joints of NOX2-deficient mice are characterized by increased neutrophil infiltration and increased expression of *Cxcl2*, *Cxcl3, Cxcl10*, and *Mmp-3*, with decreased surface expression of PD-L1 [132]. This led to the conclusion that ROS may play a protective role depending on the stage of immune activation. Accordingly, in a CIA mouse model, inhibition of NOX2 aggravated arthritis, although during immune priming, the levels of NCF1 were markedly increased, resulting in excessive ROS generation [133].

#### Neutrophil cell death in RA

##### Apoptosis in RA

An important feature of peripheral blood and synovial fluid neutrophils in patients with RA is delayed apoptosis through the increased activity of NF-κB. Gene expression analysis of RA neutrophils revealed increased mRNA levels of antiapoptotic proteins such as MCL-1 (Table 1) and decreased expression of proapoptotic proteins such as caspase-9 [49, 134]. This delay in apoptosis may be driven by increased levels of the inflammatory cytokines G-CSF, GM-CSF, IL-1β, TNF, IFN-α and IFN-γ in the synovial fluid [134–137]. However, in vivo studies have yielded conflicting results regarding the involvement of these cytokines in the inhibition of apoptosis. In addition to proinflammatory cytokines, adenosin and lactoferrin have been implicated in suppressing neutrophil apoptosis [138, 139]. Notably, when synovial fluid neutrophils from patients with RA are incubated with RA synovial fluid, they undergo apoptosis despite high levels of MCL-1. However, when mimicking hypoxic conditions in vivo, the apoptosis of RA neutrophils is delayed through the upregulation of hypoxia-inducible factor-1 (HIF-1) [140]. Indeed, HIF-1α activates NF-κB, thereby promoting cell survival [141, 142].

Several studies using murine models of arthritis have highlighted the critical role of efferocytosis in modulating disease activity. In a CIA mouse model, inhibition of the macrophage receptor T-cell immunoglobulin and mucin-domain containing 4 (TIM-4), which recognizes the ‘eat-me’ signal phosphatidylserine on apoptotic neutrophils, worsened arthritis in the early stages of disease. In contrast, blocking TIM-4 during established disease attenuated inflammation, as confirmed in a CAIA mouse model. Mice lacking Mer tyrosine kinase (MERTK), a macrophage receptor involved in efferocytosis, presented increased accumulation of apoptotic cells and developed synovial joint inflammation. Similarly, deletion of the *Axl* gene worsened arthritis. However, a recent publication using *Tyro3*, *Axl* and *Mertk* KO mice revealed contrasting findings on cytokine production and joint inflammation. *Tyro3* deficiency resulted in improved arthritis and a decrease in inflammatory cytokine levels, whereas *Axl* or *Mertk* KO aggravated disease severity [143].

Arthritic mice (K/BxN) deficient in engulfment and cell motility protein (ELMO1), a key regulator of cytoskeleton rearrangement in efferocytosis, presented reduced disease severity, reduced bone damage and reduced recruitment of neutrophils. Moreover, in a CIA mouse model, the inhibition of Rac1, which acts downstream of ELMO1, also resulted in decreased inflammation [144, 145]. Signal regulatory protein-α (SIRPα) on macrophages interacts with CD47 on neutrophils to regulate phagocytosis and migration. Treatment of STIA mice with a SIRPα agonistic antibody reduced neutrophil numbers in synovial fluid and lowered arthritis scores [146].

##### Ferroptosis in RA

Elevated levels of ferrous iron have been detected in the synovial fluid of patients with RA and are correlated with DAS28 scores (Table 1) [147]. Consistent with these findings, ELISA analysis of peripheral blood samples from patients with RA revealed decreased levels of glutathione, GPX4 and other proteins involved in regulating ferroptosis [148]. Extensive lipid peroxidation was observed in neutrophils from the synovial fluid of patients with RA, potentially due to decreased activity of the antioxidant enzyme GPX4 [149]. Ferroptosis of neutrophils is associated with NET release, triggering further inflammation [150].

##### Necrosis and necroptosis in RA

Primed human neutrophils and synovial fluid neutrophils harvested from patients with RA express cytoplasmic fibroblast activation protein-α (FAP-α), a serine protease involved in the activation of phosphoinositide 3-kinase (PI3K). This occurs in parallel with the CD44-mediated activation of PI3K through the RIPK3–MLKL–p38 axis. Both pathways result in NOX2-dependent ROS production, ultimately leading to neutrophil necroptosis [151]. IFN-γ was found to inhibit necroptosis in CIA mice, since knockout of IFN-γ was associated with increased levels of MLKL, RIPK1, and RIPK3, along with more severe joint damage and hyperinflammation [152].

##### Pyroptosis in RA

Increased serum levels of IL-18 and caspase-1 have been observed in patients with RA (Table 1) [153]. While the mRNA and protein levels of the NOD-like receptor family, pyrin domain containing 3 (NLPR3) inflammasome, NLRP3 and apoptosis-associated speck-like protein containing a CARD (ASC) are decreased in peripheral blood neutrophils from patients with RA, caspase-1 levels are increased. In addition, caspase-1 levels were positively correlated with DAS28 scores and with IL-18 levels, whereas no correlation was detected with IL-1β levels. These findings suggest an NLRP3-independent upregulation of IL-18 [154]. However, whole-blood intracellular protein expression assays revealed increased basal protein levels of NLRP3, ASC, caspase-1 and pro-IL-1β in patients with active RA, with the levels of IL-1β being increased by TLR3/4-mediated signaling. Furthermore, caspase-1 or caspase-8 inhibition reversed the TLR3/4-mediated production of IL-1β [155]. IL-18 primes neutrophils, upregulates the surface expression of CD11b and formyl peptide receptor 1 (FPR1; the receptor for formylated peptides), increases the level of intracellular calcium, activates the p38 MAPK signaling pathway and induces ROS production and NE release [156]. This finding suggests a potential role for IL-18, which is released by pyroptosis, in sustaining further neutrophil inflammation.

##### Autophagy in RA

Neutrophils isolated from the synovial fluid of patients with RA presented increased expression of microtubule-associated protein 1 light chain 3 (LC3), a well-established marker of autophagosome formation. Increased autophagosome levels were also detected in the circulating granulocytes of patients with RA and correlated positively with C-reactive protein (CRP) values, DAS28 scores and levels of TNF [157]. Autophagy in RA patients was further confirmed via transmission electron microscopy and detection of autophagic vacuoles. Moreover, several cytokines and chemokines that are elevated in the synovial fluid of patients with RA (e.g., IL-6, IL-10, CCL2 and CXCL8) are potential inducers of neutrophil autophagy [158]. Interestingly, neutrophil autophagy can be inhibited in vitro by the use of chloroquine (CQ) or miRNAs that silence the *ATG5* gene [158].

### Atherosclerosis

Cardiovascular diseases (CVDs) collectively remain the leading cause of death worldwide. These conditions arise from a complex interplay between genetic predispositions and multiple modifiable risk factors, among which elevated low-density lipoprotein cholesterol (LDL-C), smoking, impaired fasting glucose, high body mass index, and arterial hypertension are the most prominent [159]. Although atherosclerosis was once regarded as a relatively static condition, it is now widely recognized as a progressing, complex disease in which chronic inflammation plays a central role. Neutrophils contribute to atherosclerotic progression in multiple manners. A recent study from the UK Biobank suggested that neutrophil-mediated inflammation is detrimental, as elevated levels of S100A12, a proinflammatory protein primarily released by neutrophils, are associated with significantly increased risks of major adverse cardiovascular events (hazard ratio 1.15; *p* < 0.0001) (Table 1) [160].

Atherosclerotic lesions are characterized by the accumulation of LDL particles within the intima (i.e., the innermost layer) of the arterial blood vessel walls. Owing to their sensitivity to oxidative modifications (e.g., through ROS production by neutrophils), these particles are prone to initiate local inflammatory responses characterized by, for example, the activation of endothelial cells and innate immune cells [161, 162]. In addition to being found in both the early and late stages of atherosclerotic disease, high numbers of neutrophils are found in rupture-prone lesions (i.e., those containing a larger lipid core, a reduced amount of collagen, increased macrophage influx and fewer smooth muscle cells) or regions with high inflammatory activity (e.g., shoulder regions of the plaque) [163–165]. Recently, Letian et al. demonstrated a central role for TLR2 signaling upstream of neutrophil lipoprotein uptake and subsequent neutrophil arrest in lipid-rich regions in TLR2-KO mice [166]. Gelatinases derived from neutrophils can contribute to plaque instability, as increased levels of matrix metalloproteinases, including MMP-9, are detected within vulnerable regions of human atherosclerotic plaques [165, 167].

Neutrophils might also contribute to the progression of atherosclerosis through the release of NETs. For example, in atherosclerosis-prone mice (*Lysm*^*gfp/gfp*^*Apoe*^*−/−*^*)* fed a high-fat diet, neutrophils that release NETs tend to adhere luminally to the carotid bifurcation. In contrast, no neutrophil adhesion or NET release was observed in mice receiving a chow diet [168]. Baratchi et al. recently reported that shear stress triggered NETosis through the mechanosensitive ion channel Piezo1. In addition, pretreatment of neutrophils with shear stress increased NETosis induced by chemical stimuli such as LPS and adenosine triphosphate (ATP) [169].

As discussed earlier, NETs are web-like structures composed of depolymerized chromatin, citrullinated histones, and cellular proteins needed for the elimination of pathogens. While the release of extracellular traps was originally described for neutrophils, multiple types of myeloid cells (e.g., eosinophils, macrophages, etc.) also appear able to produce extracellular traps. In addition, in *Ldlr*^*−/−*^ mice with hematopoietic cell–specific deletion of DNase1 and DNase1L3, endoplasmic reticulum stress reduces DNAse secretion by plaque macrophages and thereby impairs NET clearance [170]. The presence of NETs may subsequently further promote local inflammation, as they may prime macrophages to release inflammatory cytokines, such as IL-1β and IL-6, as previously described in APOE/PR3/NE^*−/−*^ mice [78]. The release of these cytokines may, in turn, further promote NETosis and thus contribute to a positive feedback loop toward inflammation [171].

However, knowledge on the role of NETs in the development and progression of atherosclerosis in patients remains incomplete or inconsistent. For example, while Pertiwi et al. reported that NETs are abundantly present within human complicated, but not intact, coronary plaque segments, Zhai et al. reported a gradual decrease in NETs with plaque progression compared with macrophage-derived extracellular traps in a model of high-fat diet-fed *Ldlr*^*−/−*^ mice [172, 173]. Released extracellular traps might activate stimulator of interferon genes - suppressor of cytokine signaling 1 (STING-SOCS1) and TLR4-MYD88 (myeloid differentiation primary response 88) signaling within surrounding vascular smooth muscle cells. TLR4 signaling results in the release of inflammatory components, including TNF, MMP-9 and MMP-12, whereas the activation of STING-SOCS1 inhibits signal transducer and activator of transcription 3 (STAT3) phosphorylation and thereby the contractility of vascular smooth muscle cells [172, 173]. Although their exact role remains to be clarified, these findings suggest that neutrophils play a central role in chronic inflammation and contribute to atherosclerosis.

### Inflammatory bowel disease (IBD)

IBD is a chronic inflammatory condition that results in dysfunction of different parts of the gastrointestinal tract. The umbrella term IBD encompasses Crohn’s disease (CD) and ulcerative colitis (UC). Moreover, both CD and UC tend to have multifactorial etiologies, including genetic predispositions, changes in the gut microbiota and environmental factors. Neutrophils are suspected to play a central role, as mucosal infiltration of neutrophils is considered a hallmark of active IBD. This finding is supported by the fact that fecal calprotectin (also known as the granule protein S100A8/9) is still the main biomarker used to monitor disease activity in a noninvasive manner (Table 1) [174]. S100A8/9 is a member of the S100 family of proteins and is expressed predominantly in innate immune cells, including neutrophils, monocytes and macrophages. Within neutrophils, it represents ~50% of all cytosolic proteins and functions as an endogenous DAMP downstream of, for example, TLR4 activation [175, 176]. In addition, the neutrophil product NGAL, which is often suggested as an alternative fecal or serum biomarker in IBD, was recently shown to induce a profibrotic phenotype in human colon fibroblasts (CCD-18Co cells) characterized by increased alpha-smooth muscle actin (α-SMA) and collagen type 1 (COL1) protein levels. Intestinal fibrosis is a frequent complication of IBD, for which no targeted therapy is available [177, 178]. The contribution of neutrophils to IBD pathogenesis is suspected to be dual in origin. Epithelial cells within the gastrointestinal mucosa produce chemokines, including the neutrophil attractants and activators CXCL5 and CXCL8. Upon reaching the site of chemokine production, neutrophils further contribute to the disruption of epithelial barrier integrity by releasing cytotoxic components, including ROS, NETs or granular proteins such as MPO, defensins and lysozymes [174, 179]. Notably, elevated levels of PAD4, a key enzyme in NETosis, have been previously reported in colonic biopsies from patients with IBD [180]. In murine models, neutrophil-derived PAD4 exacerbates mucosal inflammation by inducing the apoptosis of intraepithelial cells. This effect is mediated by the citrullination of mitochondrial creatine kinase 1, which decreases its stability and subsequently compromises mitochondrial homeostasis [181]. In line with these findings, a recent study established a significant correlation between MPO activity in feces and the endoscopic severity of IBD [182]. Moreover, histological examination of colon tissue derived from patients with UC revealed a more pronounced neutrophil-dominated inflammatory pattern in those who were resistant to corticosteroid therapy than in those who were sensitive [183]. In addition, researchers analyzing gene coexpression patterns in inflamed IBD tissue have identified a consistent gene signature, referred to as M4–M5, in patients who do not respond to anti-TNF therapy. By making use of in silico cell type deconvolution, they demonstrated that the M4 and M5 signatures are predominantly associated with stromal cells and granulocytes [184]. Interestingly, high levels of oncostatin M (OSM), a member of the IL-6 family of cytokines that are expressed mainly by neutrophils in the circulation, correlate with a poor response to anti-TNF therapy in patients with IBD [185, 186]. However, similar to those in RA, neutrophils might also play some protective roles, as neutrophil depletion in murine animal models may aggravate colitis [187]. For example, CD177^+^ neutrophils tend to play a protective role in IBD. Compared with CD177^-^ neutrophils, CD177^+^ subsets produce lower levels of proinflammatory cytokines (i.e., IFN-γ, IL-6, and IL-17A) but higher levels of IL-22 and TGF-β and show increased bactericidal activities (i.e., ROS, antimicrobial peptides and NETs). In addition, mice deficient in CD177 develop more severe dextran sulfate sodium (DSS)-induced colitis [188].

Ongoing clinical trials focusing on neutrophils in IBD include a phase IV trial (NCT06626165) that determines the effectiveness of the IL-23 inhibitor mirikizumab in patients with strong neutrophilic infiltration of the colonic mucosa (NCT06626165). Similarly, a phase III study with the IL-23 receptor antagonist icotrokinra was initiated in patients with moderate to severe UC (NCT07196748). IL-23 plays an essential role in the terminal differentiation of IL-17-producing T effector cells. IL-17 is considered a key pathogenic cytokine in T-cell-mediated autoimmunity and stimulates innate immunity through the upregulation of granulocyte-colony stimulating factor (G-CSF) [189]. Murine and human studies have previously suggested that IL-23-treated neutrophils might selectively produce IL-17 [e.g., through upregulation of the transcription factor RAR-related orphan receptor gamma (RORγ)]. However, the production of IL-17 by neutrophils has been investigated by other researchers, who have questioned neutrophil purity by sequencing experiments or the specificity of antibodies via immunohistochemistry [190–192].

Other ongoing clinical trials include NCT07184996/NCT07185009 and NCT07080034, which investigate the efficacy of Duvakitug® and BCD-261, respectively, in patients with moderately to severely active UC. Both candidate drugs are monoclonal antibodies that target TNF-like protein 1 A (TL1A), which binds to death receptor 3 (also known as decoy receptor 3 (DR3)), thereby inducing proinflammatory downstream signaling (e.g., through the activation of MAPK and NFκB (NCT07184996, NCT07185009 and NCT07080034)). DR3 is predominantly expressed on lymphocytes but indirectly influences neutrophil physiology. In murine models, the use of an agonistic antibody targeting DR3 exacerbated DSS-induced colitis and increased the intestinal infiltration of neutrophils (e.g., by stimulating the production of GM-CSF) [193]. Similarly, other ongoing clinical trials of anti-CCR9, anti-integrin (α4β7), anti-TNF or tyrosine kinase inhibitors will undoubtedly have effects on neutrophil physiology (NCT06029972, NCT06290934, NCT06975722, NCT06681324, and NCT04338204).

A growing but largely unexplored field of interest is the interaction between microbiota or microbiota-derived factors and innate immunity, specifically neutrophils [174, 194]. Although there is disagreement on the potential existence of disease-specific microbiome signatures, the link between altered microbiome states and chronic inflammation is undeniable. Studies have shown a significant reduction in commensal gut microbiota diversity within IBD patients. However, many studies provide correlations/associations rather than causality [195]. Among the metabolites produced by the microbiota, and of particular interest during inflammation, are short-chain fatty acids (SCFAs), including formate, acetate, propionate and butyrate. SCFAs function as potential energy sources, as they can be metabolized by entering the tricarboxylic acid cycle after conversion to acetyl-CoA or succinyl-CoA. In addition, they play crucial roles in regulating both adaptive and innate immunity [196]. For example, rat-derived neutrophils show reduced production of proinflammatory mediators, including TNF, when treated with SCFAs [197]. Moreover, in a C57BL/6 mouse model of DSS-induced colitis, the application of SCFAs (butyrate) attenuated intestinal inflammation and was associated with increased expression of M2 macrophage-associated proteins, suggesting a contribution to the polarization of M1 macrophages toward the M2 phenotype [198]. Others reported significant inhibitory effects of butyrate on the in vitro protein production of IL-6, TNF, IFN-γ, LCN2 and S100A8/A9 by neutrophils from patients with IBD (at the mRNA and protein levels) [199].

### Systemic lupus erythematosus (SLE)

SLE is a relapsing-remitting chronic autoimmune disease that affects several organ systems with multifaceted clinical symptoms ranging from mild rashes (e.g., butterfly rash) to life-threatening organ damage. The global prevalence is estimated to be ~3.4 million, with an annual incidence of 400,000 new cases worldwide. The predisposition for SLE consists of a complex combination of genetic, epigenetic, hormonal, and environmental factors. In general, the influence of genetics seems to be linked mainly to children and male individuals, with genes involved being divided into immune-stimulating, immune-signaling and debris clearance genes. Immune-stimulating genes are referred to as ‘IFN signature’ genes, with several single nucleotide polymorphisms (SNPs) linked to SLE development found in IFN regulatory factor (IRF) 5 and IRF7. Genetic variants in the MHC class I and II genes, especially HLA-DRB1, are the main immune signaling genes, whereas impaired debris clearance is associated with genetic alterations in the genes encoding C1q and C4 of the complement system. Frequently, the presence of autoantibodies, including antinuclear autoantibodies (ANAs), antiphospholipids (aPLs), anti-β2-glycoprotein (aβ2GPI), anti-double-stranded DNA (anti-dsDNA) and anti-Smith (anti-Sm) autoantibodies, is observed in SLE patients. Among these, the latter appear highly specific. Immune complexes containing these autoantibodies and complement factors are deposited throughout several organ systems, resulting in the typical disease complications associated with SLE, such as vasculitis, rash, arthritis, and lupus nephritis [200].

#### NETs in SLE

The release of NETs is a key process in the pathology of SLE, as spontaneous NETosis is observed in neutrophils isolated from patients with SLE (Table 1) [201]. NETs could be used as potential biomarkers correlated with disease activity, disease flares and, although controversial, renal manifestations [202–204]. In addition, three NET-related genes, HMGB1, integrin-β superfamily member 2 (ITGB2), and cAMP-responsive element binding protein 5 (CREB5), were recently proposed as diagnostic biomarkers for SLE [205].

Several autoantibodies targeting NET components, such as MPO, high mobility group 17 (HMG-17), catalase, NE and citrullinated, methylated or acetylated histones, are elevated in sera of patients with SLE. In this disease, immune complexes act as primary triggers of NETosis [44]. The incubation of neutrophils from healthy donors with the plasma of patients with SLE induced neutrophil activation through the recognition of RNA-bound immune complexes by TLR8 [206]. The expelled NET debris is subsequently taken up by plasmacytoid dendritic cells (pDCs), which function as antigen-presenting cells that activate T and B cells. Type I IFNs, which are produced by pDCs and immune complex-derived RNA and DNA fragments, can activate neutrophils [44]. Recently, LDNs isolated from patients with SLE were shown to form complexes with platelets in a TLR7-dependent manner, resulting in increased NET formation [207]. In contrast to LDNs, which exhibit a hyperinflammatory phenotype in SLE patients, NDNs from patients with SLE presented decreased CD66b, increased production of CXCL10, increased release of MMP-8 and decreased NET release [208].

Like in RA, in addition to increased NET production, impaired NET clearance contributes to SLE pathogenesis [201]. This can be the consequence of defective DNase-I activity because of genetic variations, increased activity of endogenous DNase-I inhibitors, shielding of NET debris by autoantibodies or the protective effect of ribonucleic acid oxidation [201, 202]. Nucleic acid oxidation not only impairs enzymatic digestion by DNases but also increases the activation of the cyclic guanosine monophosphate-adenosine monophosphate synthase (cGAS)–STING pathway in macrophages, resulting in increased IFN production [209].

Although counterintuitive at first glance, intravenous administration of the neutrophil attractant and activator CXCL5 to lupus-prone MRL/lpr (*Faslpr)* mice improved survival and was characterized by reduced NET release and reduced neutrophil proliferation, activation and recruitment. However, by increasing CXCL5 levels in circulation, the chemotactic gradient toward neutrophil attractants in tissue may be destroyed and potentially even reversed, resulting in reduced tissue infiltration. Additionally, in the peripheral blood of patients with SLE, CXCL5 levels are reduced, and intravenous administration of CXCL5 can reverse this imbalance [210].

#### ROS in SLE

Circulating immune complexes in the serum of patients with SLE can induce an oxidative burst in healthy neutrophils [211, 212]. These complexes activate neutrophils through FcγRII and subsequent Abelson murine leukemia viral oncogene homolog 1 (ABL1) and Rous sarcoma oncogene (SRC) signaling [213]. The resulting ROS production, together with degranulation, causes direct tissue damage and thereby contributes to the development of, for example, glomerulonephritis [214]. In addition to direct cytotoxicity, oxidative stress influences adaptive immunity. It skews T cells toward a proinflammatory phenotype, mainly Th17 cells, and reduces the Treg cell population [215]. In vitro assays revealed the binding of galectin-1 (Gal1) to V-set and transmembrane domain-containing 1 (VSTM1), thereby regulating neutrophil viability and ROS production. However, Gal1 mainly occurs in its oxidized form in the circulation of patients with SLE, likely due to a decrease in reduced glutathione. The oxidized Gal1 is unable to bind VSTM1, resulting in the loss of this inhibitory pathway, as shown in neutrophils harvested from the peripheral blood of patients with SLE (Table 1) [216].

However, other researchers highlighted the protective effect of ROS on the development of SLE. Impairment of NOX2 in the *Ncf1m1J* mutant mouse model resulted in impaired ROS production, increased expression of IFN type I-regulated genes and increased levels of autoantibodies [217]. These findings were validated in SLE patients carrying a missense mutation in the *NCF1* gene [218].

#### Neutrophil cell death in SLE

In contrast to the delayed neutrophil apoptosis in RA, neutrophil death is enhanced in SLE and results in extensive release of autoantigens into the extracellular environment. Increased apoptosis in neutrophils is consistent with elevated levels of FasL and TRAIL in the serum, whereas the levels of GM-CSF appear to be significantly lower (Table 1). Interestingly, supplementation of SLE serum with GM-CSF resulted in decreased neutrophil apoptosis in vitro [54, 201, 219]. Neutrophil apoptosis in SLE is mediated by the activity of caspase-8 and caspase-9, as demonstrated in vitr*o* by the use of specific inhibitors [54]. IgG antibodies isolated from patients with SLE bind FcγRIII and subsequently induce apoptosis by increasing the expression of FasL in neutrophils originating from MRL/lpr SLE mice [220]. In patients with juvenile-onset SLE, nuclear self-antigens are exposed on the cell surface of apoptotic neutrophils, thereby activating TLR3, TLR8 and TLR9 on peripheral blood mononuclear cells (PBMCs), thus initiating an IFN-α inflammatory response [221, 222].

Inefficient clearance of apoptotic neutrophils also contributes to SLE pathogenesis. SLE macrophages presented reduced CD44 expression and reduced phagocytic capacity, which correlated with antinuclear autoantibodies and disease activity. Increased levels of soluble TAM receptors (MERTK, TYRO3 and AXL) are detected in the serum of patients with SLE, supporting the hypothesis of impaired clearance. Furthermore, when apoptotic cells are not efficiently cleared, a phenomenon called ‘secondary necrosis’ occurs, releasing DAMPs into the extracellular environment. These DAMPs trigger inflammation and autoantibody production [54, 223]. In patients with disease flare-ups, apoptotic microparticles containing elevated levels of acetylated histones induce NETosis [224]. In addition to NETosis, apoptotic microparticles originating from the plasma of patients with SLE drive the release of IL-6, TNF and IFN-α by plasmacytoid and myeloid dendritic cells from healthy donors [225].

With respect to pyroptosis, in vitro incubation of neutrophils from healthy donors with SLE serum induced FcRγ-mediated activation of caspase-1 and caspase-11, which cleave gasdermin D into its active form, gasdermin D-N. This molecule forms complexes in the cellular membrane, releasing mtDNA into the extracellular environment. The oxidized mtDNA, in turn, was shown to induce gasdermin D‒N pore formation [226].

Combined data from SLE human neutrophils and in vivo mouse SLE models revealed a critical role for GPX4 in regulating neutrophil ferroptosis. IFN-α and IgGs from patients with SLE induce the cAMP response element modulator-α (CREM-α)-regulated decrease in the transcription of GPX4 [227].

Although therapeutic interventions do not target neutrophils directly, they do affect innate immunity. Whole-blood transcriptomics and plasma proteomics data from a phase III clinical trial with anifrolumab, an antibody that blocks the receptor for type I interferons, revealed that this treatment downregulated apoptosis and NETosis-related pathways in neutrophils and improved neutropenia [228]. Belimumab, which targets BAFF, has extensive effects on the innate immune system, diminishing neutrophil numbers and neutrophil activation. Furthermore, neutrophil degranulation is a predictive marker of the response to belimumab treatment [229]. Mesenchymal stem cell (MSC) therapy has also emerged as a promising approach for treating SLE. The administration of MSCs to MRL/lpr mice and to patients with SLE induces an extracellular vesicle storm, which originates from bone marrow neutrophils. TNF causes neutrophil aggregation in the bone marrow, which results in the ICAM-1/Rab11b-mediated release of extracellular vesicles, as shown in vitro in neutrophils from the respective KO mouse models. This resolves the imbalance between Th17 and Treg cells in the SLE mouse model through the LILRB4/STAT5/STAT3 axis [230].

### Chronic obstructive pulmonary disease (COPD)

COPD is a chronic respiratory disease caused by exposure to inhaled noxious particles, among which tobacco smoke and air pollutants are the most important. COPD is the third leading cause of death globally. The pathogenesis of COPD is heterogeneous and frequently associated with emphysema, chronic inflammation of the airways, extensive mucus production, and vascular dysfunction. Pulmonary emphysema is a pathological and permanent dilatation of the distal airways (i.e., bronchioles, alveolar ducts and alveoli) due to loss of elasticity in the lung parenchyma. Loss of elastic recoil within the alveoli may result in rupture, disrupting the alveolar‒capillary surface and impairing effective gas exchange. COPD is characterized by periods of acute worsening, which are defined as “exacerbations”. The occurrence of exacerbations is associated with acceleration of the underlying disease and increased morbidity and mortality. Treatment strategies for COPD include pharmacological (e.g., bronchodilators and anti-inflammatory inhalation therapy such as corticosteroids) and nonpharmacological approaches (e.g., exercise training, smoking cessation). Currently, no curative treatment strategy for COPD is available [231, 232].

Although the exact underlying pathophysiology remains incompletely understood, chronic inflammation plays a crucial damaging role. Macrophages and neutrophils are believed to be crucial in COPD pathogenesis. As such, in murine models (C57BL/6 J and FVB/N mice), cigarette smoke inhibits the clearance of apoptotic cells (i.e., efferocytosis), including dead neutrophils (Table 1) [233]. While increased levels of neutrophils and CXCL8 are found in patients with stable COPD, neutrophil counts, as well as levels of CXCL8, NE and CCL5, are significantly increased during exacerbations [234–237]. While other researchers also reported a correlation between NE and disease severity [i.e., staged according to the Global Initiative for Obstructive Lung Disease score (GOLD score)], they did not find a correlation with exacerbations [238]. Indeed, many current studies focusing on exacerbations describe correlations rather than clear causalities. Inflammation associated with exacerbations results in the release of multiple chemokines/cytokines, including CXCL8, and may hereby induce multiple para-phenomena, such as neutrophil activation. As correlations may be influenced by multiple confounding factors, this might result in contradictory findings. Currently, there are no validated biomarkers available to predict COPD prognosis or risk for exacerbations [231, 239]. Similarly, Bafadhel et al. defined four different exacerbation clusters on the basis of potential biological biomarkers of 145 patients with COPD: (1) a proinflammatory exacerbation endotype characterized by high concentrations of airway TNF and IL-1β, (2) a T1 inflammatory phenotype with high concentrations of airway NK cell and T-cell attractants and activators CXCL10 and CXCL11, (3) a T2 inflammatory phenotype with high concentrations of airway CCL17 and IL-5 and (4) a pauci-inflammatory phenotype with low concentrations of measured airway biomarkers [239]. The existence of these different immunophenotypes may be an important confounding factor to keep in mind when initiating clinical trials for COPD. This finding may provide an explanation for why candidate drugs, such as MEDI8968, which targets IL-1 receptor 1, or AZD9668, which targets neutrophil elastase, failed to reduce the rates of exacerbations [240–242]. Although primarily focusing on bronchiectasis instead of COPD (patients with respiratory symptoms driven mainly by COPD were excluded), a phase III trial with brensocatib [an oral reversible inhibitor of dipeptidyl peptidase 1 (dpp-1), which has a principal role in the activation of serine proteases, including NE and cathepsin G] recently revealed significantly fewer pulmonary exacerbations than [243]. Upon submission of this manuscript, a phase Ib trial with XH-S004 (another oral DPP-1 inhibitor) in patients with COPD is planned to start recruitment (NCT07035652). Other ongoing clinical trials targeting neutrophil activation in COPD include a phase II trial (NCT05270525) with nebulized ensifentrine. Ensifetrine blocks phosphodiesterases 3 and 4 (PDEs 3 and 4, with the latter predominantly expressed in neutrophils), which results in increased amounts of cyclic adenosine 3′,5′-monophosphates (cAMP/cGMP) and thereby anti-inflammatory signaling. For example, cAMP-mediated activation of protein kinase A (PKA) induces the production of proresolving factors through activation of the nuclear transcription factor CREB. Additionally, PKA may also suppress proinflammatory pathways, including NFκB and PI3K signaling [244].

In 2011, the REACT study showed positive results with the use of another oral PDE4 inhibitor, roflumilast, as add-on therapy to the best-available inhaled therapies. However, *post hoc* analysis of the REACT data indicated that roflumilast was most effective at reducing the incidence of exacerbations (moderate or severe) among patients hospitalized for COPD in the previous year [RR 0.771, 95% CI 0.610–0.974; *p* = 0.029] [245, 246]. This subgroup analysis shows comparable results when applied to the dataset of a similar clinical trial, RE^2^SPOND, which failed to report significant differences in patients treated with roflumilast as add-on therapy [245, 247].

#### Neutrophil enzymes in COPD

Neutrophils are generally known to contribute to COPD pathogenesis and progression through multiple mechanisms. One of these mechanisms involves degranulation and the release of proteolytic enzymes, such as NE. Released proteases might either directly (e.g., breakdown of elastin) or indirectly (e.g., proteolytic activation of the macrophage elastase MMP-12) contribute to the modification of the extracellular matrix within lung tissue [239]. Increased amounts of the MMP-12 protein have been described in the serum of patients with COPD compared with healthy controls (Table 1). MMP-12 mRNA levels in PBMCs from patients with COPD correlated with the severity of airflow limitation. However, no difference was observed at the protein level [234]. In addition to MMP-12, MMP-9 levels are dose-dependently increased in bronchoalveolar lavage fluid from BALB/c mice exposed to cigarette smoke for four days [248]. Increased levels of MMP-9 in the serum of patients with COPD, which are negatively correlated with the forced expiratory volume in one second (FEV1), are also well documented. Although an increase in the concentration of MMP-9 may suggest increased proteolytic activity, the net proteolytic activity is more appropriately reflected by the ratio of MMP-9 to its inhibitor TIMP-1. With respect to the MMP-9/TIMP-1 ratio in patients with COPD, the results of current studies are controversial [249–251].

By using a mouse model of COPD (based on C57BL/6J mice), researchers recently showed that the inhibition of NE by GW311616A (a specific NE inhibitor) attenuated airway inflammation and mucus overproduction and prevented the decrease in lung function induced by chronic exposure to cigarette smoke. Moreover, inhibition of NE prevents NETosis induced by cigarette smoke extract by blocking the nuclear translocation of NE and thereby chromatin decondensation [252]. Similarly, in a mouse model of elastase-induced COPD-like changes, treatment with GW311616A reduced the pathological features of virus-induced exacerbations. As such, the inhibition of NE diminishes the concentrations of total/citrullinated nucleosomes and reduces both histological inflammation scores and the concentrations of chemokines (e.g., CXCL10 and CCL5) and proinflammatory cytokines (e.g., TNF, IL-1β and IL-6) in bronchoalveolar lavage fluid [253]. Interestingly, studies in cGAS^−/−^/TLR9^−/−^ mice revealed that NET-derived DNA promotes NF-κB-dependent inflammation through cGAS and TLR9. The activation of these receptors results in the upregulation of inflammatory cytokines, including TNF and IL-1β [254]. The amount of NETs within the sputum of patients with stable COPD correlates with the GOLD score and its individual parameters, annual exacerbation frequency, and predicted FEV1 and CAT scores (i.e., a COPD assessment test, which is a questionnaire used to quantify the impact of COPD on a person’s life) [238].

In addition to the attributed effects described above, NE might have more indirect mechanisms of action in COPD, for example, through the regulation of gene expression. By using a transgenic mouse model called IKTA mice (i.e., mice with doxycycline-inducible expression of constitutively active human IKK-β in the airway epithelium), researchers have shown that neutrophilic inflammation during lung development disrupts elastic fiber assembly and induces a COPD-like lung phenotype specifically within the saccular stage of development. In addition, purified human NE appeared to inhibit the mRNA expression of elastic fiber assembly components in lung fibroblasts during the saccular stage of development [255].

### Fibrotic diseases (e.g., interstitial lung fibrosis, myelofibrosis)

The presence of chronic inflammation may result in a dysregulated tissue repair response and consequently fibrogenesis (i.e., the deposition of fibrotic tissue). Although fibrosis has a multifactorial, complex etiology and the exact role of neutrophils often remains speculative, there is increasing evidence for a contributing role of neutrophil-mediated inflammation in fibrogenesis.

For example, patients with idiopathic lung fibrosis (IPF) have increased concentrations of S100A9 in bronchoalveolar lavage fluid, which is positively correlated with the number of neutrophils (Table 1) [256]. Moreover, increased amounts of CXCL8 are found within the serum and bronchoalveolar lavage fluid of patients with IPF and are negatively correlated with both markers of disease severity (e.g., gas transfer and lung function) and survival [257–259]. A role for CXCL8 in fibrogenesis has also been suggested by authors focusing on other diseases associated with fibrosis and chronic inflammation, such as endometriosis and myelofibrosis (discussed further) [260–262].

Increased amounts of NE and NETs have been reported in the BALF and lung tissue sections of patients with IPF, and both components promote the differentiation of lung fibroblasts into myofibroblasts [263–266]. Compared with fibroblasts, myofibroblasts exhibit contractile properties and are characterized by enhanced production of an extracellular matrix, which contributes to tissue stiffening. The contractile properties of myofibroblasts are mediated through the expression of α-SMA, an actin protein present in vascular smooth muscle cells. The development of myofibroblasts can be considered a functional cell fate in response to tissue injury. In fact, multiple cell types have been identified as potential precursor cells of myofibroblasts. Among these cells are adipocytes, pericytes, smooth muscle cells and multipotent mesenchymal stromal or stem cells [267, 268]. Myofibroblast activation can occur through multiple mechanisms, including mechanical (e.g., loss of structural integrity) and biochemical stimuli, which were recently extensively reviewed by Younesi et al. [267]. TGF-β, one of the most studied profibrotic growth factors, is secreted as a dimer and occurs in 3 isoforms, of which TGF-β1 is the most prevalent. Under normal circumstances, TGF-β is predominantly present in its latent form through (non)covalent interactions with proteins such as latent TGF-β binding proteins (LTBP), which facilitate storage within the extracellular matrix [267]. Through interaction with its receptors, TGF-β induces the activation of canonical signaling via the phosphorylation of suppressors of mothers against decapentaplegic (SMAD) proteins. This further induces the expression of profibrotic genes, including α-SMA. In addition to canonical signaling, TGF-β may act noncanonically through other pathways, including the mitogen-activated protein kinase (MAPK), NF-κB and Rho-associated kinase (ROCK) pathways [269]. Neutrophils might contribute to TGF-β bioavailability through multiple mechanisms, including the release of proteases (e.g., MMPs and NEs) or the production of ROS and RNS [270–272]. Indeed, treatment with the NE inhibitor sivelestat in C57BL/6 mice with bleomycin-induced fibrosis prevented fibrosis. In these mice, sivelestat significantly inhibited the formation of active TGF-β1 and phosphorylated SMAD2, whereas total TGF-β1 remained unaffected [270]. In addition to being expressed by neutrophils, PDE4 is expressed in lung fibroblasts and is crucial for TGF-β-induced differentiation into myofibroblasts [273]. These findings provide a rationale for ongoing clinical trials with PDE4 inhibitors [e.g., HSK44459 (NCT06764862, phase II) and nerandomilast (NCT06238622, phase III)] in patients with IPF.

Proteolysis of structural extracellular matrix proteins by enzymes, including MMPs, results in the release of matricryptins, which subsequently may induce a positive feedback loop that further stimulates fibrogenesis and/or inflammation. Matricryptins are peptides that vary in size from a few to hundreds of amino acids and interact with receptors of multiple cell types (e.g., integrins and growth factor receptors), including fibroblasts and neutrophils [274]. In vitro studies have shown that MMP-9 promotes latent TGF-β activation and induces α-SMA expression in fibroblasts (MRC-5) [271, 275]. Traditionally, MMP-9 is secreted in the form of pro-MMP-9 (also known as zymogen), in which the zinc ion within the catalytic site is linked by a sulfhydryl bond to a cysteine within the prodomain. The activation of pro-forms can be achieved by proteolytic removal of the pro-domain or by chemical modification, increasing the accessibility of the catalytic site, for example, by ROS/RNS [276, 277]. Interestingly, MMP-9 is particularly released by neutrophils, and more specifically, its isoform is free of tissue inhibitor of metalloproteinases (TIMP). The TIMP family of proteins contains 4 isoforms (TIMP-1 to -4), which form noncovalent 1:1 complexes with MMPs, resulting in their inhibition. While TIMPs show overlapping abilities to inhibit MMPs, TIMP-1 preferentially binds MMP-9 [276, 278]. In addition to the release of matricryptins and the activation of TGF-β, MMP-9 is suspected to contribute to fibrosis by increasing the bioavailability of vascular endothelial growth factor (VEGF). Although incompletely understood, one of the mechanisms might be the local release of VEGF stored within the extracellular matrix by local degradation [279]. Increased angiogenesis, especially in the early stages, is observed in fibrotic diseases, including liver, lung and myelofibrosis (discussed further) [280–282].

The release of matricryptins by proteolysis has pleomorphic effects. As such, the collagen-1-derived matricryptin proline-glycine-proline (PGP) can attract and activate neutrophils through interactions with the receptors CXCR1/2. In mice, intratracheal delivery of MMP-8/MMP-9 together with prolyl endopeptidase (PE) results in PGP, the amount of which is correlated with polymorphonuclear/neutrophil influx. PE is a serine protease that performs specific cleavage at the C-terminal side of a proline, where it is able to produce PGP from the “PPGP” motif present in collagen. Interestingly, the generation of PGP appears dependent on PE activity and vice versa, as neither MMP-8/MMP-9 nor PE can induce PGP production on their own [274, 283]. Interestingly, concentrations of PGP within the sputum of patients with COPD correlate positively with disease severity. Treatment with azithromycin, a macrolide antibiotic with anti-inflammatory properties that is frequently used as a chronic therapy for COPD, significantly reduces sputum PGP concentrations and is associated with a reduced exacerbation frequency. In contrast to other authors, this study did not report statistically significant differences in MMP-9 levels between patients treated with azithromycin or placebo [284, 285]. In addition to PGP, fibulin-1 peptide 1 (FBLN1C1, amino acids 567-586) is another matricryptin with a suggested role in fibrogenesis, as its levels are reported to be increased in the serum and lung tissue samples of patients with IPF [286, 287]. Fibulin-1 is a matrix glycoprotein that is incorporated within the extracellular matrix and facilitates its stabilization. In vitro studies have shown that FBLN1C1 increases the viability and proliferation of pulmonary fibroblasts derived from patients with IPF [288]. One of the proposed mechanisms by which FBLN1C1 induces fibrosis involves its binding with latent TGF-β-binding protein 1 (LTBP1), thereby allowing the activation of TGF-β1 and the initiation of TGF-β signaling through the phosphorylation of downstream SMAD proteins [289].

Myelofibrosis (MF) is a rare hematological disorder characterized by the progressive deposition of fibrotic tissue within the bone marrow and is a type of malignancy called “*BCR::ABL1-*negative myeloproliferative neoplasm (MPN)”. Generally, patients with primary myelofibrosis (PMF) are distinguished from patients who develop myelofibrosis secondary to another MPN (postessential thrombocythemia MF and post-polycythemia vera MF). Patients with MF have a complicated disease course often characterized by progressive anemia, symptomatic splenomegaly (due to extramedullary hematopoiesis) and constitutional symptoms. Depending on the underlying risk factors, median overall survival (OS) varies from several years for low-risk patients to several months for individuals in high-risk groups. The most frequent causes of death include leukemic transformation, progressive disease with bone marrow failure and complications from thrombosis [290]. Patients with MPN frequently carry mutations in driver genes, including Janus kinase 2 *(JAK2*), myeloproliferative leukemia virus oncogene *(MPL*) and calreticulin (*CALR*), resulting in uncontrolled activation of the inflammatory JAK-STAT pathway [291]. In addition, MPNs are characterized by increased cytokine expression patterns in both peripheral blood and bone marrow and are therefore considered a model of inflammation-related malignancies [292–294]. Although direct evidence remains limited, multiple factors suggest an underlying role of neutrophils in MPN pathophysiology. As such, patients with high absolute or relative [defined as the neutrophil‒lymphocyte ratio (NLR)] neutrophil counts at diagnosis have a more severe prognosis and higher mortality rates [295]. For a more extensive overview of the role of neutrophils in MF, we refer to a recent review article [296]. Neutrophils might contribute to disease progression through various mechanisms, including the release of ROS or proteases. Indeed, neutrophils from the peripheral blood of patients carrying mutated JAK2 show increased ROS-producing capacity compared with neutrophils from healthy controls [297]. As mentioned earlier, redox imbalances contribute to the activation of TGF-β through, for example, interactions with latency-associated protein (LAP), to which TGF-β is noncovalently bound [272, 298]. Moreover, in line with other fibrotic diseases, the concentrations of neutrophil-derived and profibrotic proteases such as NE and MMP-9 are increased within the peripheral blood of patients with MF [299, 300]. In addition, neutrophils carrying the most prevalent driver mutation, JAK2V617F, exhibit increased expression of CD24. CD24^high^ neutrophils exhibit increased GM-CSF-JAK2-STAT5-dependent signaling and evade efferocytosis. Moreover, genetic loss of CD24 or antibody blockade tends to reduce the emperipolesis of neutrophils by megakaryocytes and the production of TGF-β [301]. Previously, researchers reported reduced emperipolesis and fibrosis in GATA1^low^ mice (i.e., a model of MF development) treated with the CXCR1/2 antagonist reparixin [302]. In line with this, a phase II clinical trial with reparixin in patients with PMF, post-ET and post-PV MF who are resistant or intolerant to standard therapy with JAK inhibitors has been initiated (NCT05835466). Emperipolesis is an intriguing type of cell‒cell interaction wherein one cell is engulfed by another without affecting the other’s viability and can occur between multiple cell types. Emperipolesis between neutrophils and megakaryocytes has been reported within healthy individuals but significantly increases in circumstances with increased hematopoietic stress, such as in systemic inflammation or MF. The exact pathophysiological consequences of emperipolesis are currently far from understood [303, 304]. However, authors recently reported interesting findings, including emperipolesis-mediated membrane transfer of intracytoplasmic neutrophils to platelets, whereas others detected increased neutrophilic mRNA transcripts within the platelets of patients with MPN [305, 306].

## Conclusion

By representing the most prevalent type of white blood cell within the human body, neutrophils play a nonnegligible role in both physiological and pathophysiological conditions. As part of the innate immune system, neutrophils have previously been investigated predominantly within the context of acute inflammatory conditions and are considered less relevant within chronic inflammatory conditions. In addition, researchers tend to focus more on other types of immune cells, as studying neutrophil characteristics is complicated by practical challenges, such as the inability to cryopreserve them and fragility to in vitro conditions. Despite the pivotal role of neutrophils in acute conditions, an increasing number of researchers are intrigued by their potential role in orchestrating tissue homeostasis and chronic inflammation. As discussed within this review, this interest is supported by the ability of neutrophils to carry out both direct and indirect inflammation modifying functions, for example, by altering the behavior of monocytes, endothelial cells, or adaptive immune cells. The pro- or anti-inflammatory effects of neutrophils are established by their various effector functions, among which are NETosis, ROS, degranulation or various types of cell death (e.g., apoptosis). However, despite significant progress in the understanding of the role of neutrophils in chronic inflammatory diseases, many questions regarding their functions in different circumstances remain. In addition, future research should focus on the development of a uniform nomenclature that defines neutrophil heterogeneity in health and disease. Further studies aiming to answer these questions will hopefully contribute to the development of new treatment strategies to prevent or modulate chronic inflammation in a context-dependent manner.
